# CD137+CD154− Expression As a Regulatory T Cell (Treg)-Specific Activation Signature for Identification and Sorting of Stable Human Tregs from *In Vitro* Expansion Cultures

**DOI:** 10.3389/fimmu.2018.00199

**Published:** 2018-02-07

**Authors:** Anna Nowak, Dominik Lock, Petra Bacher, Thordis Hohnstein, Katrin Vogt, Judith Gottfreund, Pascal Giehr, Julia K. Polansky, Birgit Sawitzki, Andrew Kaiser, Jörn Walter, Alexander Scheffold

**Affiliations:** ^1^German Rheumatism Research Centre (DRFZ) Berlin, Leibniz Association, Berlin, Germany; ^2^Miltenyi Biotec GmbH, Bergisch Gladbach, Germany; ^3^Department of Cellular Immunology, Clinic for Rheumatology and Clinical Immunology, Charité – University Medicine, Berlin, Germany; ^4^Institute for Medical Immunology, Charité – University Medicine, Berlin, Germany; ^5^Department of Genetics/Epigenetics, Saarland University, Saarbrücken, Germany; ^6^Berlin-Brandenburg Center for Regenerative Therapies (BCRT), Charité – University Medicine, Berlin, Germany

**Keywords:** regulatory T cells, chimeric antigen receptor, adoptive regulatory T cell therapy, regulatory T cell stability, regulatory T cell expansion, regulatory T cell signaling, CD137

## Abstract

Regulatory T cells (Tregs) are an attractive therapeutic tool for several different immune pathologies. Therapeutic Treg application often requires prolonged *in vitro* culture to generate sufficient Treg numbers or to optimize their functionality, e.g., *via* genetic engineering of their antigen receptors. However, purity of clinical Treg expansion cultures is highly variable, and currently, it is impossible to identify and separate stable Tregs from contaminating effector T cells, either *ex vivo* or after prior expansion. This represents a major obstacle for quality assurance of expanded Tregs and raises significant safety concerns. Here, we describe a Treg activation signature that allows identification and sorting of epigenetically imprinted Tregs even after prolonged *in vitro* culture. We show that short-term reactivation resulted in expression of CD137 but not CD154 on stable FoxP3+ Tregs that displayed a demethylated Treg-specific demethylated region, high suppressive potential, and lack of inflammatory cytokine expression. We also applied this Treg activation signature for rapid testing of chimeric antigen receptor functionality in human Tregs and identified major differences in the signaling requirements regarding CD137 versus CD28 costimulation. Taken together, CD137+CD154− expression emerges as a universal Treg activation signature *ex vivo* and upon *in vitro* expansion allowing the identification and isolation of epigenetically stable antigen-activated Tregs and providing a means for their rapid functional testing *in vitro*.

## Introduction

Adoptive transfer of regulatory T cells (Tregs) represents an attractive approach to exploit physiological self-regulating capacities for prevention or treatment of immune-mediated pathologies such as graft-versus-host disease (GvHD), organ transplantation, or chronic inflammatory diseases. Transfer of polyclonal Tregs, either *ex vivo* (1, 2) or after *in vitro* expansion, has been shown to be safe and effective for prevention of GvHD (3–8). In autoimmune diseases Treg treatment also seems to be safe, but therapeutic efficiency has so far not been sufficiently demonstrated (9–13). Essentially, within polyclonal Treg populations, the number of Tregs with therapeutically relevant specificity may be too small to achieve optimal clinical effects. This might be overcome by increased Treg doses or alternatively *via* selection of Tregs with disease-relevant specificities. Indeed, experimental models have demonstrated increased therapeutic potential of antigen-specific Tregs compared to polyclonal Tregs, e.g., by targeting disease-relevant autologous or allogeneic antigens in type 1 diabetes (T1D) (14–17), GvHD (18–25), experimental autoimmune encephalomyelitis (EAE) (26, 27), and arthritis (28, 29). However, generation of antigen-specific Tregs and thus their therapeutic application is currently limited by their low frequencies, limited knowledge about the identity of disease-relevant target antigens, and lack of technologies for antigen-specific Treg selection and expansion. Therefore, genetic engineering has been used to redirect antigen-specificity of human Tregs using transgenic T cell receptors (TCRs) (30–32) or chimeric antigen receptors (CARs). The immunosuppressive potential of CAR–Tregs, which can be universally applied to all donors independent of matched MHC alleles, has been shown to prevent development of EAE (33), colitis (34–36), GvHD (37–39), allergic airway inflammation (40), and neutralizing immune responses against Factor VIII (41) in mice. Most importantly, improved Treg-based therapies largely depend on efficient technologies for the *in vitro* expansion and manipulation of their functional properties. However, *in vitro* cultured Tregs display highly variable purities resulting from contaminating effector T cells (Teffs) or potential Treg instability. So far, there are no markers for the rapid identification and sorting of stable Tregs from such expansion cultures. To date, FoxP3 expression and above all demethylation of a Treg-specific demethylated region (TSDR) within the FoxP3 locus represent the gold standard for estimating the fraction of stable Tregs within a population (42–45), yet both do not allow for sorting of the specific subset.

In particular for Tregs equipped with disease-relevant antigen receptors, e.g., autoantigens, the risk to generate unpredictable numbers of Teffs with disease-amplifying potential has to be tightly controlled. However, the lack of discriminative markers also affects systematic functional optimization of *in vitro* generated Tregs, e.g., by genetic engineering. For example, transgenic TCR or CAR constructs may need to fulfill different requirements in Tregs *versus* Teffs, which is currently difficult to test in mixed cultures without clear-cut discriminative markers. Thus, the lack of markers for the identification of stable Tregs represents a major obstacle for the generation of expanded and functionally optimized Tregs for clinical applications.

A number of Treg-specific, activation-induced surface markers, such as CD137 (46–48), CD121a/b, LAP, GARP (49–51) or Ox40/CD39 (52), have been described to identify activated Tregs *ex vivo*. Among those, CD137 is expressed after only 5–7 h of antigenic stimulation and has been proven to be highly specific for Tregs (46, 47), allowing their *ex vivo* discrimination from CD137−CD154+ Teffs. CD137 expression enabled the specific enrichment of antigen-activated Tregs *ex vivo*, displaying all features of thymic Tregs such as a demethylated TSDR and a Treg-specific expression profile, including high levels of FoxP3, Helios, CTLA4, and lack of CD127 and effector cytokines (46, 47). After polyclonal stimulation of Tregs *ex vivo*, Schoenbrunn et al. further demonstrated that co-staining of CD137 and CD154 allowed further enrichment of stable Tregs by exclusion of T cells co-expressing both markers (48). Whether this Treg signature is also maintained after activation and expansion *in vitro* and still allows discrimination from instable Tregs or Teffs are not known but would strongly improve current possibilities for optimal *in vitro* expansion of Tregs. Here, we show that after brief polyclonal or antigen-specific stimulation, CD137+CD154− expression represents a universal Treg-specific activation signature for the identification and sorting of stable, TSDR demethylated Tregs after prior *in vitro* expansion.

## Materials and Methods

### Treg Isolation

Leukapheresis products from healthy donors were obtained from the Charité University Hospital, Berlin, Germany, with informed consent according to ethical guidelines. PBMCs were obtained by Ficoll-Paque (GE Healthcare Life Sciences, Freiburg, Germany) gradient centrifugation. CD25+ Tregs were isolated from PBMCs according to manufacturer’s recommendations using CD25 microbeads (Miltenyi Biotec, Bergisch Gladbach, Germany). Tregs were cultured in “Treg expansion medium” consisting of TexMACS medium (Miltenyi Biotec, Bergisch Gladbach, Germany) + 5% (v/v) human AB-serum (Sigma-Aldrich, Schnelldorf, Germany) + 100 U/ml IL-2 + 100 nmol rapamycin (both Miltenyi Biotec, Bergisch Gladbach, Germany) and 100 U/ml penicillin/100 μg/ml streptomycin (Gibco^®^, Thermo Fisher Scientific, Schwerte, Germany) in the presence of Treg expansion beads (Miltenyi Biotec, Bergisch Gladbach, Germany) at a bead-to-cell ratio of 4:1. During expansion, fresh culture medium was added every 2–3 days.

### Dextran (Dex)–CAR Generation

Dextran-specific CAR–Tregs with varying extracellular spacer domains were generated using lentiviral vectors encoding for a PGK promoter-driven AC146-derived single-chain variable fragment (scFv) (vh/vl orientation) linked to a human IgG4 hinge (L, M, XS) (53) or a human CD8 hinge (S) in combination with a CD8 transmembrane domain and the intracellular signaling modules of CD137 and CD3ζ. Additional lentiviral constructs shared the same scFv, an XS spacer, and the CD8 transmembrane region but differed with regard to their costimulatory and signaling domains consisting of either CD3ζ or CD3ε without any costimulation or in combination with ICOS, CD28, CD134, CD137, or PD-1. All constructs contained a P2A-linked ΔLNGFR. Lentiviral supernatants were generated by co-transfection of HEK293T cells with the expression vector and packaging plasmids. One day prior to transfection, 3 × 10^6^ HEK293T cells were seeded in a 10-cm cell culture dish in complete DMEM (cDMEM) consisting of DMEM (Gibco^®^), + 10% FCS + 100 U/ml penicillin, 100 µg/ml streptomycin + 50 µM 2-Mercaptoethanol (all Thermo Fisher Scientific, Schwerte, Germany). Cells were transiently transfected with 0.84 µg pMDG-2.VSV-G, 5.16 µg pCMVΔR8.74, and 3.35 µg Dex–CAR plasmids diluted in ddH_2_O supplemented with 2.5 M CaCl_2_. While aerating, 2 ml of 2× HBS buffer (136.89 mM NaCl, 4.96 mM KCl, 1.76 mM Na_2_HPO_4_, 20.98 mM HEPES in ddH_2_O, pH = 6.75–6.76) were slowly added to the solution, and 2 ml of the transfection solution was added dropwise to the cells. The medium containing the transfection solution was removed after 4 h, and cells were washed twice with prewarmed PBS before fresh cDMEM was added. After 48 h, lentiviral supernatants were harvested, filtered (0.45 µm), and used directly or stored at −80°C for up to 6 months.

### Treg Transduction and Activation

CD25+ Tregs were isolated and cultured as described above, and on d3 culture medium was replaced with the respective lentiviral supernatants supplemented with 4 µg/ml protaminsulfate (Sigma-Aldrich, Schnelldorf, Germany). Cells were spinoculated on 96-well plates coated with retronectin (Takara Bio via Clontech Laboratories, Saint-Germain-en-Laye, France) for 90 min at 800 × *g* and 32°C. After centrifugation, viral supernatant was removed, and “Treg expansion medium” was added to the cells. Untransduced Tregs and CAR–Tregs were expanded for 10–12 days, and medium was replaced every 2–3 days. Tregs were rested for 2 days without magnetic bead particles in RPMI-1640 (Gibco^®^, Thermo Fisher Scientific, Schwerte, Germany) + 5% (v/v) human AB-serum (Sigma-Aldrich, Schnelldorf, Germany) + 100 U/ml penicillin/100 μg/ml streptomycin (Gibco^®^, Thermo Fisher Scientific, Schwerte, Germany) before 6 h restimulation with Treg expansion beads (4:1 bead-to-cell ratio, Miltenyi Biotec, Bergisch Gladbach, Germany), soluble FITC Dex (MW: 2,000,000, 2 µg/ml, Sigma-Aldrich, Schnelldorf, Germany), bead-bound Dex (1:100; Dex-coated microbeads in PBS, Miltenyi Biotec, Bergisch Gladbach, Germany), or 10 ng/ml PMA and 500 ng/ml ionomycin (Sigma-Aldrich, Schnelldorf, Germany). For cytokine staining, 5 µg/ml Brefeldin A (Sigma-Aldrich, Schnelldorf, Germany) were added for the last 4 h of stimulation. 1 µg/ml anti-CD40 antibodies (Miltenyi Biotec, Bergisch Gladbach, Germany) were added to the stimulation when CD154 was stained on the surface. When expression was analyzed together with cytokines, intracellular staining of CD137 and CD154 was performed.

### Flow Cytometry

Cells were stained in different combinations with the following antibodies according to manufacturer’s recommendations: CD4-PE-Vio770, CD4-APC-Vio-770, CD4-FITC, CD4-VioBlue (VIT4), CD25-VioBright FITC (4E3), CD127-FITC, CD127-PE-Vio770 (MB15-18C9), CD271 (LNGFR)-PE, CD271 (LNGFR)-PE-Vio770 (ME20.4-1.H4), CD137-PE (4B4-1), CD154-APC, CD154-VioBlue (5C8), HLA-A2 (REA517), TNF-α-PE-Vio770 (CA2), IL-2 APC-Vio770 (N7.48 A), IL-17-FITC (CZ8-23G1), FoxP3-APC (3G3) (all Miltenyi Biotech, Bergisch Gladbach, Germany), CD25-BV421 (BC96), and IFN-γ-PerCP Cy5.5 (4S.B3; both from Biolegend, San Diego, CA, USA). Viobility 405/520 Fixable Dye (Miltenyi Biotech, Bergisch Gladbach, Germany) or propidium iodide (Sigma-Aldrich, Schnelldorf, Germany) were used to exclude dead cells. Intracellular cytokine staining was performed using the Inside Stain Kit (Miltenyi Biotec, Bergisch Gladbach, Germany), and intracellular FoxP3 staining was performed using the FoxP3 Staining Buffer Set (Miltenyi Biotec, Bergisch Gladbach, Germany) all according to manufacturer’s protocol. For staining of CAR surface expression, Tregs were incubated for 10 min with 2 µg/ml FITC-labeled Dex (MW: 2,000,000, Sigma-Aldrich, Schnelldorf, Germany) at 4°C together with labeling of other surface molecules. All data were acquired on a FACS Canto/LSRII (BD, Heidelberg, Germany) or MACS Quant Analyzer (Miltenyi Biotec, Bergisch Gladbach, Germany), and FACS sorting was performed on an Aria I, Aria II, or Influx Cell Sorter (BD, Heidelberg, Germany). FlowJo (TreeStar, Inc, Ashland, OR, USA) was used for data analysis.

### Quantification of Gene Expression

The competitive expansion of Dex–CAR constructs with different signaling domains was analyzed by quantitative real-time PCR. DNA was isolated by Zymo Research *Quick*-DNA™ Miniprep Kit (Zymo Research, Freiburg, Germany) according to manufacturer’s instructions, and gene expression was analyzed using 1× SYBR^®^ Green PCR Master Mix (Thermo Fisher Scientific, Schwerte, Germany) and 500 nmol forward and reverse primers (TIB MOLBIOL, Berlin, Germany; Table S1 in Supplementary Material), respectively. Gene expression was analyzed on a StepOne™ Real-Time PCR System (Thermo Fisher Scientific, Schwerte) and normalized to expression of GAPDH.

### Methylation-Sensitive TSDR Real-time PCR

Genomic DNA was isolated with the QIAamp DNA Mini Kit (Qiagen, Hilden, Germany) and between 50 and 1,200 ng were used for bisulfite treatment (EpiTect, Qiagen, Hilden, Germany). Real-time PCR was performed in a final reaction volume of 20 µl with 10 µl FastStart Universal Probe Master (ROX, Roche Diagnostics, Mannheim, Germany), 100 ng Lamda DNA (NEB, Frankfurt a.M., Germany), 5 pmol methylation or non-methylation specific probe, 30 pmol methylation or non-methylation specific primers, and at least 15 ng bisulfite-treated DNA or plasmid standard. Samples were analyzed in triplicates on an ABI 7500 Cycler (Thermo Fisher Scientific, Schwerte). The percentage of FoxP3 TSDR was calculated by dividing the non-methylated copy number by the total genomic FoxP3 copy number.

### Deep Bisulfite Amplicon Sequencing

CD137+CD154− and CD137+CD154+ Tregs (CD25+CD127−CD45RO+) from male donors were sorted and pooled, and cell pellets were digested with lysis buffer (10 mM Tris, 5 mM EDTA, pH 8.0) with 1 mg/ml Proteinase K (Sigma-Aldrich, Schnelldorf, Germany) at 55°C overnight. Cell lysates were used directly for bisulfite conversion of 100 ng DNA, which was treated with EZ-DNA methylation Gold kit (Zymo Research, Freiburg, Germany) according to manufacturer’s instructions. Amplification of bisulfite-treated DNA was done by PCR, which was performed with either 2.5 U HOT FIREPol^®^DNA Polymerase (Solis BioDyne, Tartu, Estonia) or 1.5 U HotStar Taq™DNA Polymerase (Qiagen, Hilden, Germany) with 20 ng bisulfite-treated DNA, 0.2 mM dNTPs, and 0.17 µM bisulfite-specific primers (Table S2 in Supplementary Material) according to manufacturer’s recommendations. Amplicons were purified with Agencourt Ampure XP beads (Beckman Coulter, Krefeld, Germany) according to manufacturer’s instructions and sequenced on the Illumina MiSeq platform using MiSeq Reagent Kit v3 (Illumina, Inc., San Diego, USA). Sequencing results were processed with BiQ Analyzer HT (54) and filtered according to sequence identity (>0.9), bisulfite conversion (>0.95), and fraction of unrecognized sites (<0.1). Data for methylation of the indicated regions in central and effector memory T cells were obtained from the study by Durek et al. (55).

### Suppression Assay

Expanded Tregs were stimulated for 6 h with Treg expansion beads (4:1 bead:cell ratio, Miltenyi Biotec, Bergisch Gladbach, Germany) before CD137+CD154− and CD137+CD154+ Tregs were sorted and expanded. After 14 days of further expansion, Tregs were rested for 2 days in RPMI-1640 medium (Gibco^®^, Thermo Fisher Scientific, Schwerte, Germany) + 5% (v/v) human AB-serum (Sigma-Aldrich, Schnelldorf, Germany) + 100 U/ml penicillin/100 μg/ml streptomycin (Gibco^®^, Thermo Fisher Scientific, Schwerte, Germany). Responder T cells (Tresps) with opposite HLA-A2 expression were isolated with the CD4^+^ T cell Isolation Kit (Miltenyi Biotec, Bergisch Gladbach, Germany) according to manufacturer’s instructions and stained with CellTrace™ Violet Cell Proliferation Kit (Thermo Fisher Scientific, Schwerte, Germany) at a final concentration of 2.5 µM. A total of 5 × 10^4^ Tresps were co-cultured with Tregs in different ratios in 96-well flat bottom plates and stimulated with Treg Suppression Inspector (Miltenyi Biotec, Bergisch Gladbach, Germany). Dilution of proliferation dye was analyzed on day 7. The percentage of inhibition was calculated as $\frac{\left(A-B\right)}{A}\times 100$, where *A* is the uninhibited Tresp and *B* is the inhibited Tresp.

### Statistical Analysis

The exact values of *n* and the respective statistical tests that were used to determine significances are specified in the respective figure legends. Statistical analysis was performed with GraphPad PRISM software 5.02 (GraphPad Inc., La Jolla, CA, USA). Significances are indicated with *p ≤ 0.05, **p ≤ 0.01, and ***p ≤ 0.001.

## Results

### *In Vitro* Expansion of CD25+ Tregs Compromises Purity

**Figure 1:**
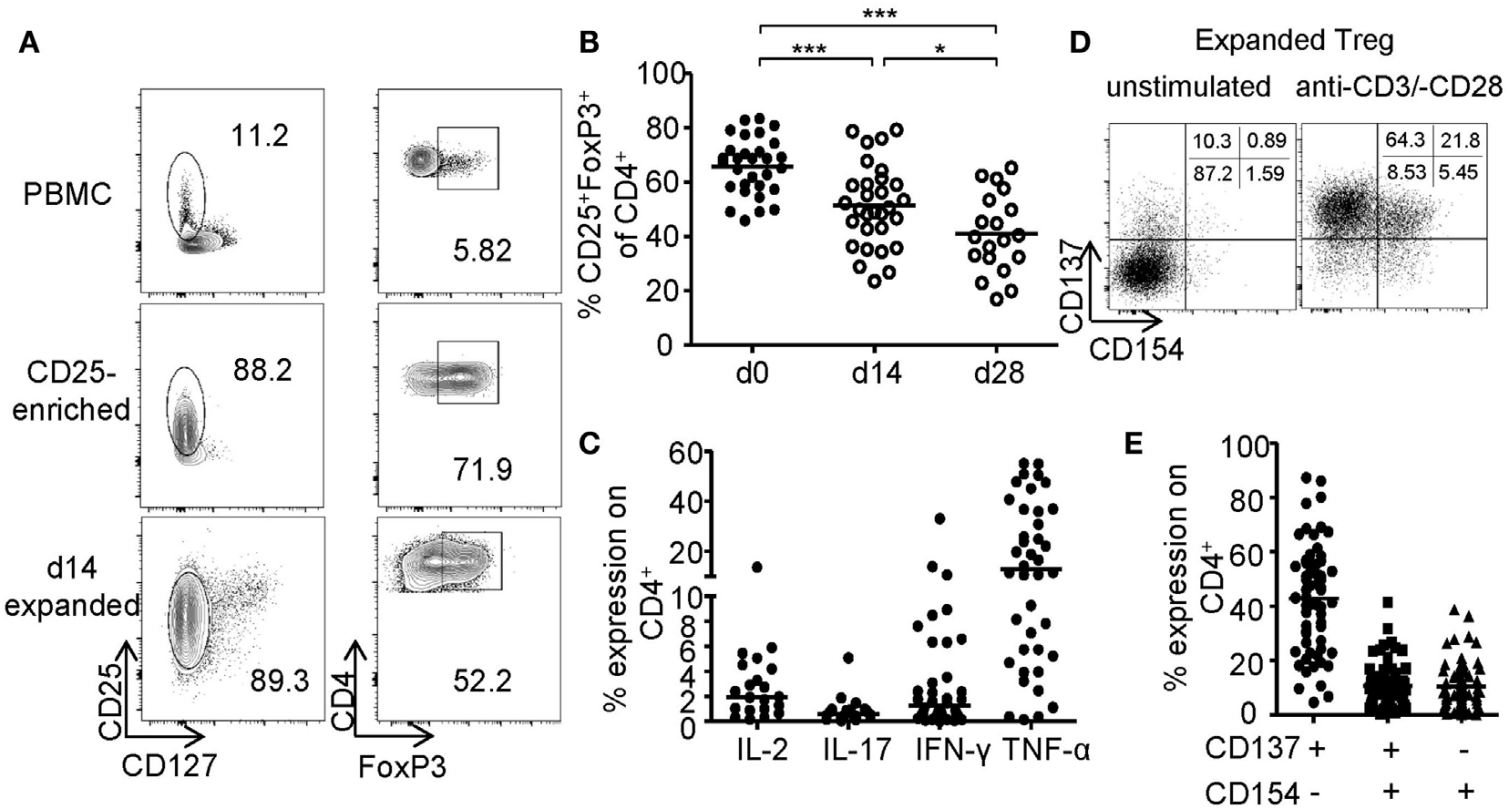
Phenotype of *in vitro* expanded regulatory T cells (Tregs). CD25+ Tregs were sorted and expanded for 14 or 28 days before analysis of **(A,B)** FoxP3 expression; **(A)** representative dot plot of one donor and **(B)** statistical analysis of several donors (*n* = 30 from nine independent experiments for d0 and d14, and *n* = 19 from seven different experiments for d28). **(C)** Cytokine expression was analyzed on d28 after 6-h restimulation with PMA/ionomycin (*n* = 38 from 12 different experiments for IFN-γ, *n* = 40 from 13 different experiments for TNF-α, *n* = 19 from 7 different experiments for IL-17, and *n* = 17 from 6 different experiments for IL-2). **(D,E)** CD137 and CD154 expression were analyzed on expanded Tregs on d14 after restimulation with anti-CD3/-CD28; **(D)** representative dot plot of one donor and **(E)** statistical analysis of several donors (*n* = 64, 21 independent experiments were performed). **(B,C,E)** Each dot represents one donor, and **(B)** statistical significances were determined by one-way analysis of variance; lines indicate **(B)** mean or **(C,E)** median.

The generation of sufficient numbers for Treg-based therapies or the modification of Treg functionality, e.g., by genetic engineering, requires prolonged *in vitro* expansion, which typically results in reduced frequencies of FoxP3+ Tregs (Figure 1A). FoxP3+ Tregs were enriched by GMP-compliant isolation using CD25 microbeads and expanded for 14–28 days using anti-CD3-/anti-CD28-coated microspheres in the presence of IL-2 and rapamycin. Upon expansion, frequencies of FoxP3-expressing cells significantly decreased resulting in cultures with only 41.01% (mean ± 14.50% SD) FoxP3+ cells after 28 days compared to 65.79% (mean ± 10.46% SD) at the beginning of the culture (Figure 1B). Furthermore, high levels of pro-inflammatory cytokines (IFN-γ, IL-17, IL-2, and TNF-α) were detected after restimulation of expanded cultures, indicating significant amounts of contaminating Teffs or potential Treg instabilities (Figure 1C). Independent of FoxP3, CD25 was expressed by almost all expanded cells, whereas CD127 expression was lost and could therefore no longer distinguish between Tregs and contaminating Teff (Figure 1A). Recently, converse expression of CD137 and CD154 has been described to discriminate between activated Tregs and Teffs *ex vivo* (46–48, 56). Therefore, we wanted to test whether these markers represent a universal activation signature, which allows discrimination between Tregs and Teffs after prolonged *in vitro* expansion. CD25+ sorted Tregs were expanded for 2 weeks as described (Figures 1A–C) and restimulated for 6 h with anti-CD3/anti-CD28. Flow cytometric analysis of CD137 and CD154 expression revealed the presence of three distinct subsets with differential CD137 and CD154 expression (Figure 1D). While most cells were defined by a CD137+CD154− phenotype, a variable percentage expressed the Teff-specific activation marker CD154 exhibiting either a CD137+CD154+ or CD137−CD154+ phenotype (Figure 1E).

### CD137+CD154− Expression Identifies Stable Tregs within Expansion Cultures

**Figure 2:**
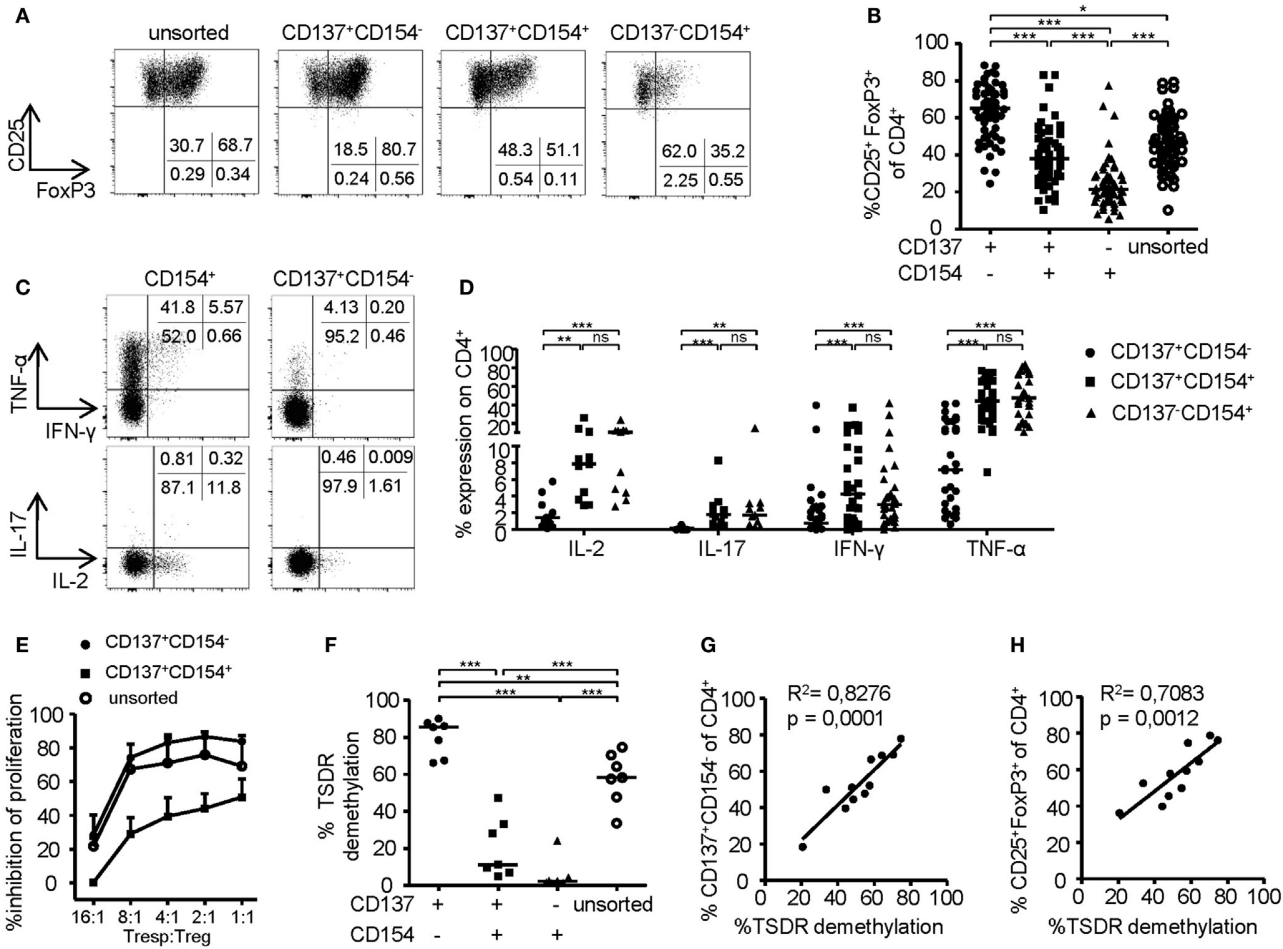
Phenotype of CD137- and CD154-expressing cells within expanded regulatory T cell (Treg) cultures. **(A,B)** CD25+ Tregs were sorted and expanded before analysis of FoxP3 expression; **(A)** representative dot plot of one donor and **(B)** statistical summary of several donors (*n* = 61, 20 independent experiments were performed). **(C,D)** CD25+ Tregs were sorted and expanded before 6-h restimulation with PMA/ionomycin for analysis of cytokine expression on CD137- and CD154-expressing cells; **(C)** representative dot plot of one donor and **(D)** statistical summary of several donors (*n* = 30 from nine different experiments for IFN-γ and TNF-α, *n* = 11 from four different experiments for IL-17 and IL-2). **(E)** Tregs were sorted from expanded CD25+ Tregs according to CD137 and CD154 expressions or left unsorted, and all populations were expanded for another 14 days before *in vitro* suppression of proliferation of CD4+CD25− effector T cells was analyzed (*n* = 4–6, two independent experiments were performed); inhibition of proliferation relative to untreated responder T cell (Tresp) is shown. **(F)** Tregs were sorted from expanded cultures according to CD137 and CD154 expression after 6-h restimulation with anti-CD3/anti-CD28 before Treg-specific demethylated region (TSDR) demethylation was analyzed (*n* = 7, two independent experiments were performed). **(G,H)** CD25-enriched Tregs were expanded for 14 or 28 days before analysis of TSDR demethylation; correlation of TSDR demethylation with **(G)** CD137+CD154− expression, and **(H)** FoxP3 expression is shown (*n* = 11, three independent experiments were performed). Statistical significances were determined by **(B,F)** Kruskal–Wallis test, **(D)** Friedman test, or **(G,H)** linear regression analysis. **(B,D,F,G,H)** Each dot represents one donor, lines indicate **(B,D,F)** median, **(E)** mean ± SEM is shown.

**Figure 3:**
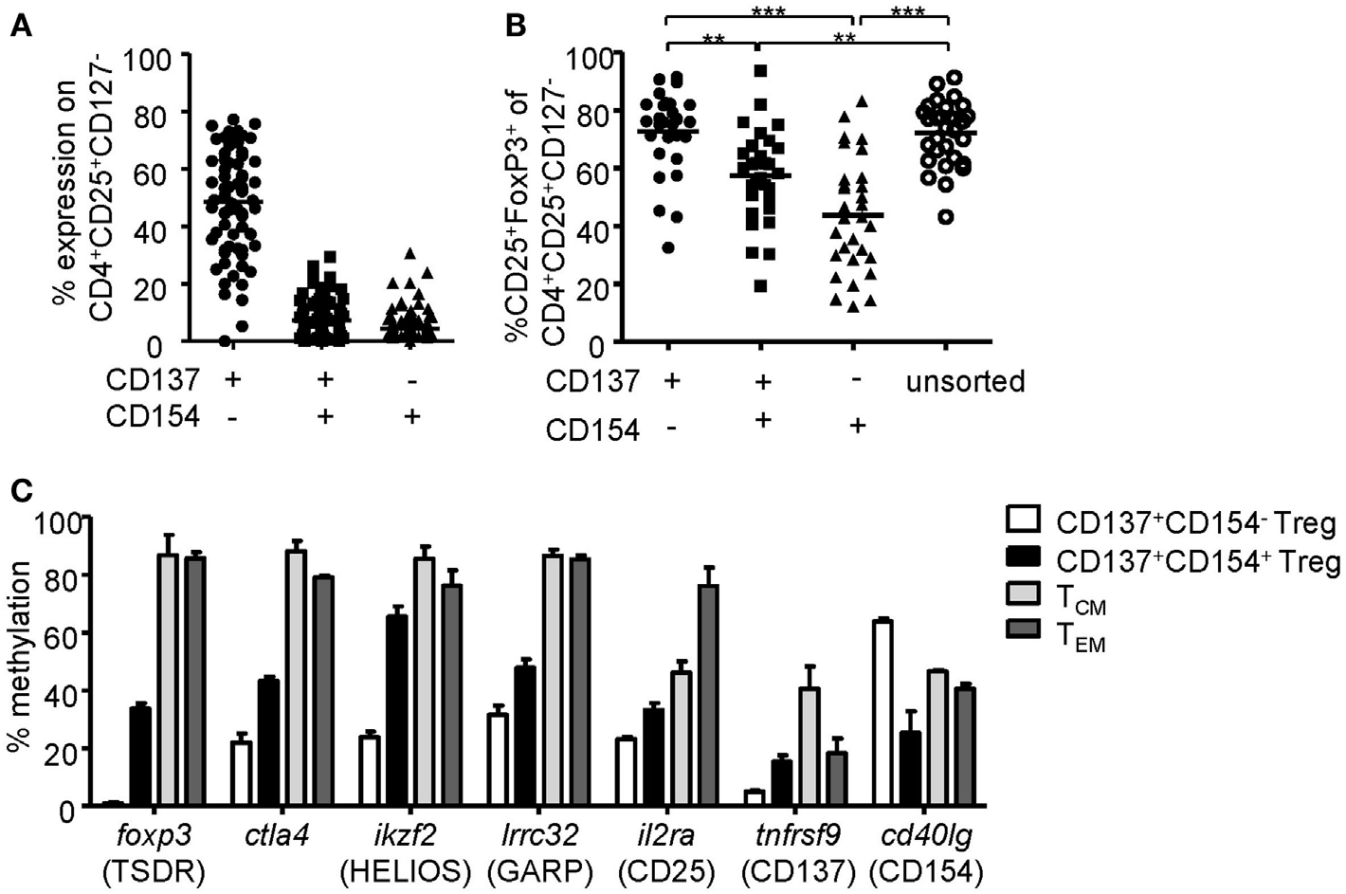
CD137+CD154− expression identifies stable regulatory T cells (Tregs) *ex vivo*. **(A)** CD137 and CD154 expression were analyzed on CD25+CD127− Tregs after 6-h stimulation with anti-CD3/anti-CD28 *ex vivo* (*n* = 68, 17 independent experiments were performed). **(B)** Frequencies of FoxP3+ cells were analyzed among CD137- and CD154-expressing cells within the CD25+CD127− Treg compartment (*n* = 30, five independent experiments were performed). **(C)** CD137+CD154− and CD137+CD154+ Tregs (CD25+CD127−CD45RO+) were sorted after 6-h stimulation with anti-CD3/anti-CD28, and methylation of indicated regions was analyzed (data from two independent experiments are shown, and five and six male donors were pooled for each experiment). Methylation of central memory and effector memory T cells was derived from the study by Durek et al. (55). **(A,B)** Each dot represents one donor, lines indicate **(A,B)** median, **(C)** mean ± SEM is shown; statistical significances were determined by **(B)** Kruskal–Wallis test.

To investigate the phenotype of CD137- and CD154-expressing cells within expanded Treg cultures, FoxP3 was stained on the different subsets after 6-h stimulation (Figures 2A,B). Remarkably, FoxP3+ Tregs were highly enriched within the CD137+CD154− Treg subset (mean ± SD, 63.24% ± 14.92), while frequencies were significantly reduced or almost completely absent within CD137+CD154+ (mean ± SD, 39.78 ± 15.76%) and CD137−CD154+ cells (mean ± SD, 24.32 ± 13.71). In addition, expression of effector cytokines was almost exclusively detected within CD154+ cells (Figures 2C,D). In contrast, CD137+CD154− Tregs completely lacked effector cytokine expression, in particular IL-2, IL-17, and IFN-γ, and they expressed only low levels of TNF-α (Figures 2C,D). Next, the *in vitro* suppressive capacities of CD137+CD154− and CD137+CD154+ Tregs were analyzed. While CD137+CD154− Tregs were highly efficient in inhibiting Teff proliferation, CD137+CD154+ Tregs exhibited an impaired suppressive potential compared to unsorted total Tregs (Figure 2E). To investigate the stability of FoxP3 expression within the different subsets, demethylation of the TSDR was analyzed revealing a striking difference between the different subsets. The TSDR was almost completely demethylated in CD137+CD154− Tregs, hypermethylated in CD137+CD154+ cells and almost completely methylated in CD137−CD154+ cells (Figure 2F). This suggests either a gradual loss of TSDR demethylation correlating with the acquisition of CD154 expression or the co-existence of Tregs and Teffs that were similarly able to express both markers. Furthermore, there was a strong association of the mean TSDR demethylation in unseparated Treg cultures with the frequency of CD137+CD154− T cells (Figure 2G), which was even stronger than correlation with the frequencies of FoxP3+ cells (Figure 2H). The different subsets that were defined by CD137 and CD154 expression were also detected within the CD25+CD127− Treg compartment *ex vivo* (Figure 3A). As observed within expansion cultures, FoxP3+ cells were significantly enriched within CD137+CD154− Tregs and strongly reduced within CD154-expressing subsets (Figure 3B). In addition to the TSDR, we also determined the methylation status of additional markers that have been shown to contribute to a stable epigenetic Treg signature and were differentially methylated between Tregs and Teffs (57, 58). CD137+CD154− Tregs exhibited an epigenetically stable Treg signature including demethylation of not only the TSDR but also *ctla4, ikf2, lrrc32, il2ra*, and *tnfrsf9*, which were almost completely methylated in central and effector memory T cells (Figure 3C). Interestingly, CD137+CD154+ Tregs exhibited an intermediate Treg–Teff epigenetic signature that further suggests that CD137+CD154+ Tregs either represent a transitional state between both subsets or a mixture of Tregs and Teffs, which have acquired the potential to co-express both markers. Thus, our data suggest that CD137+CD154− expression represents a highly specific activation signature allowing to dissect Treg populations with different suppressive potential and epigenetic stability. This activation signature enables the rapid identification and sorting of epigenetically stable FoxP3+ Tregs *ex vivo* and within expanded cultures.

### CD137 Expression Enables Rapid Analysis of Treg Activation

**Figure 4:**
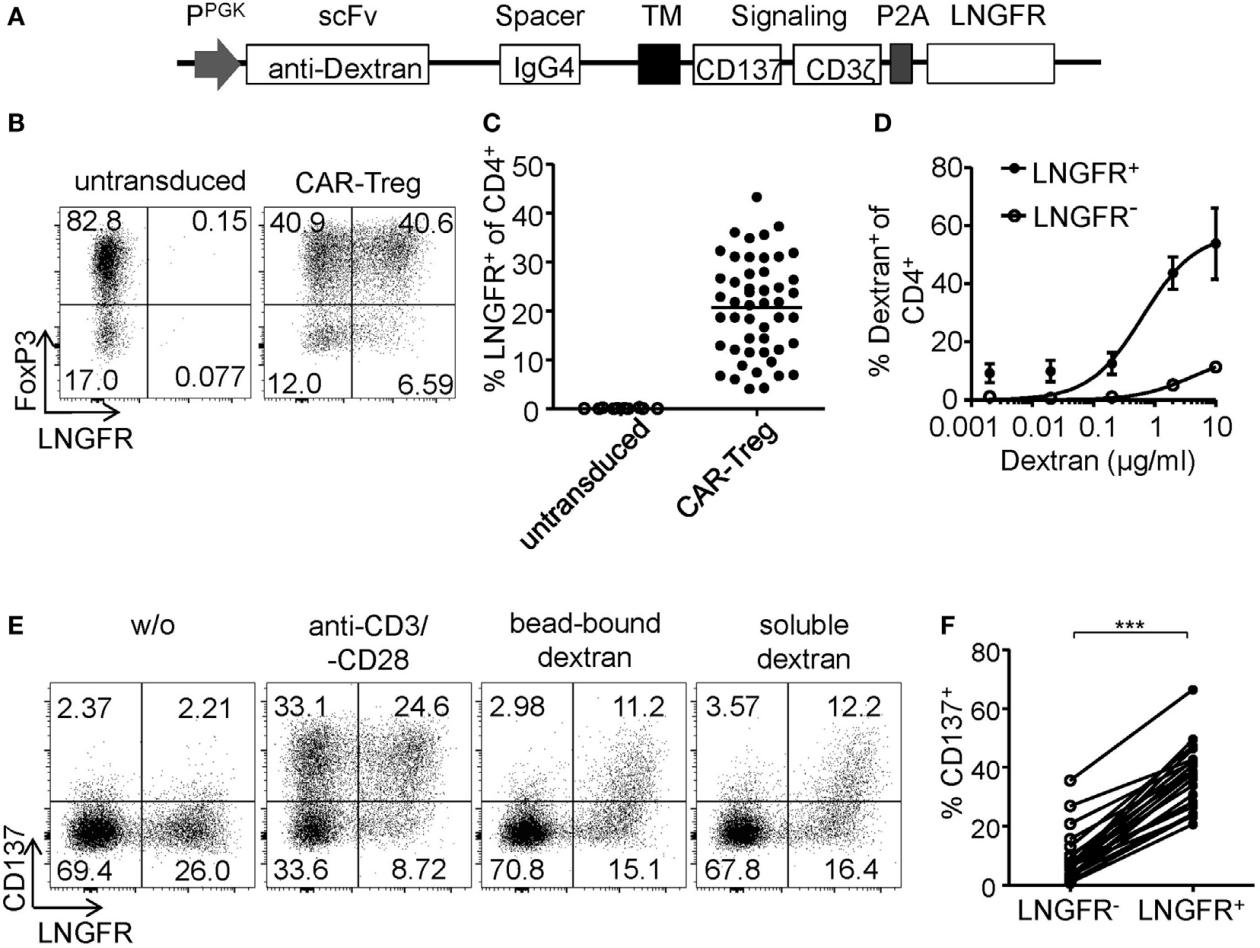
CD137 expression identifies antigen-activated chimeric antigen receptor (CAR)–regulatory T cells (Tregs). **(A)** Schematic diagram of the dextran-specific CAR construct [transmembrane [TM], 2A peptide (P2A)]. **(B–D)** CAR–Tregs were generated by lentiviral transduction, and efficiencies were analyzed by **(B–C)** LNGFR expression; **(B)** representative dot plot of one donor and **(C)** statistical analysis of several donors is shown (*n* = 50 from 16 independent experiments of CAR–Tregs and *n* = 12 from 4 different experiments of untransduced Tregs are shown). **(D)** CAR surface expression was analyzed on LNGFR+ and LNGFR− cells by incubation with FITC-labeled dextran (*n* = 3–9 from 1 to 3 independent experiments are shown). **(E,F)** Tregs were restimulated for 6 h with anti-CD3/anti-CD28, bead-bound dextran or 2 µg/ml soluble FITC dextran, and expression of CD137 was analyzed. **(E)** Representative dot plots of one donor and **(F)** statistical analysis of bead-bound stimulation are shown (*n* = 21, seven different experiments were performed). **(C,F)** Each dot represents one donor, and **(C)** lines indicate mean; **(D)** mean ± SEM is shown. **(F)** Statistical significance was determined by paired *t*-test.

**Figure 5:**
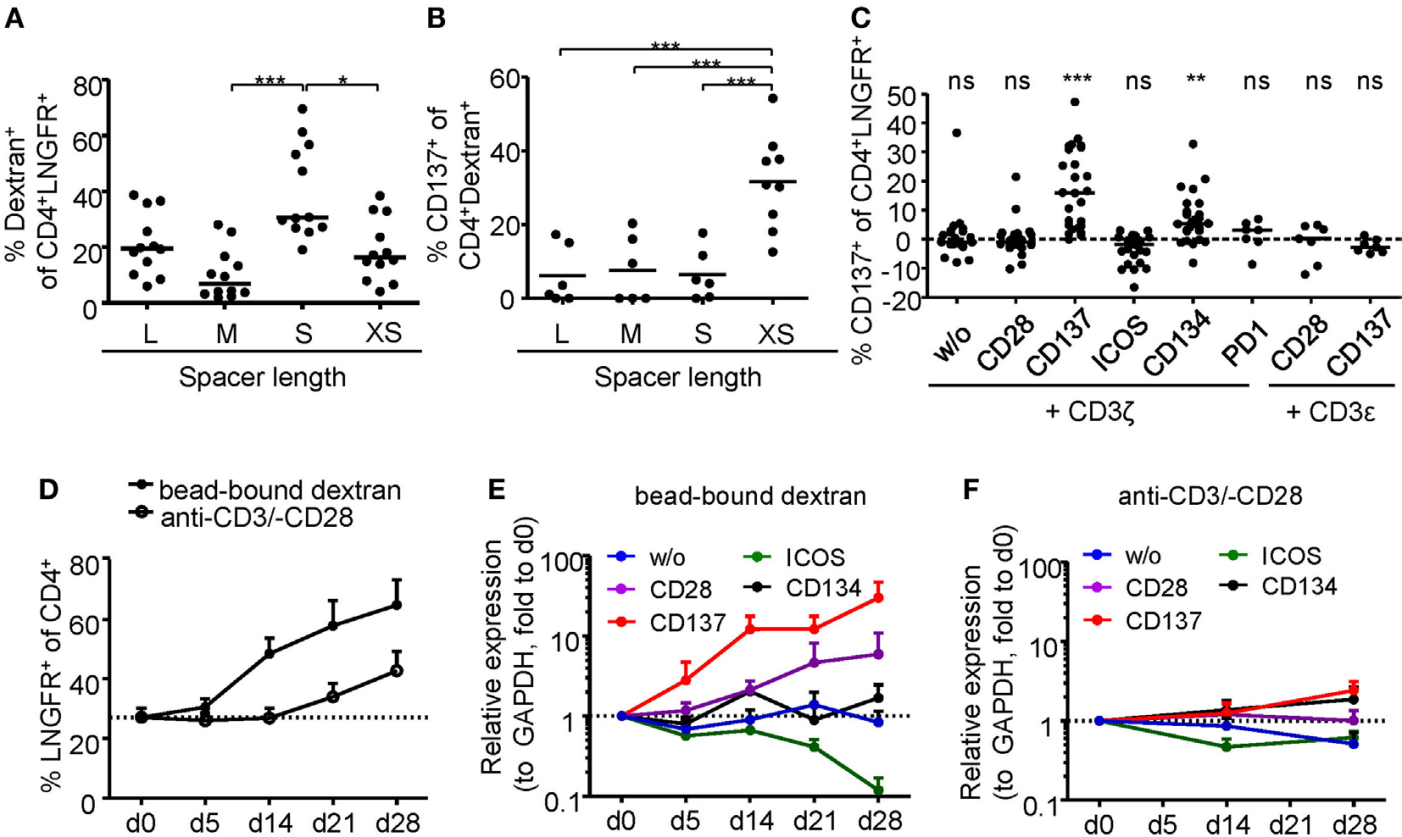
Comparison of regulatory T cell (Treg) activation by different chimeric antigen receptor constructs. **(A–B)** CD25-enriched Tregs were transduced with dextran (Dex)–CAR constructs with different spacer lengths (L = 228aa, M = 119aa, S = 45aa, XS = 12aa), and **(A)** binding of FITC-labeled Dex was analyzed (*n* = 12, four independent experiments were performed). **(B)** CAR–Tregs were restimulated for 6 h with 2 µg/ml FITC-labeled Dex, and CD137 expression was analyzed on Dex-binding cells; CD137 expression in unstimulated samples was subtracted, and negative values were set to 0 (*n* = 6–9, and two to three independent experiments were performed). **(C–F)** CD25-enriched Tregs were transduced with Dex–CAR constructs with different costimulatory domains combined with CD3ζ or CD3ε. **(C)** CD137 expression was analyzed on CAR–Tregs after 6-h restimulation with bead-bound Dex, and CD137 expression in unstimulated samples was subtracted (*n* = 22–26, seven to eight different experiments were performed). **(D–F)** CAR–Tregs with different signaling domains were pooled and expanded in the presence of anti-CD3/anti-CD28 or bead-bound Dex; **(A)** LNGFR expression was analyzed at different time points (*n* = 5–7, two to three different experiments were performed). **(E,F)** Expression (relative to GAPDH) of the different signaling domains with CD3ζ was determined at different time points of expansion with **(E)** bead-bound Dex or **(F)** anti-CD3/anti-CD28. Expression was quantified by qPCR and normalized to relative expression on d0 (*n* = 7, three different experiments were performed). Statistical significances were determined by **(A)** Kruskal–Wallis test, **(B)** one-way analysis of variance, or **(C)** Wilcoxon signed-rank test indicating activation above background. **(A–C)** Each dot represents one donor, lines indicate **(A,C)** median, or **(B)** mean; **(D–F)** mean ± SEM is shown.

Regulatory T cell functionality is dependent on activation by the antigen receptor, and quality of the TCR signal has a major impact on their suppressive potential. However, so far it has been difficult to directly determine TCR activation of Tregs due to a lack of suitable markers. In particular, in mixed cultures of stable and instable Tregs or Teffs, the clear-cut assignment of certain functional readouts to stable Tregs has been impossible since commonly used parameters such as cytokine production are limited to Teffs and instable Tregs. Genetic engineering of Treg antigen specificity represents one important example for functional optimization of Tregs, e.g., for therapeutic purposes. However, it has so far not been possible to optimize artificial antigen receptor constructs for Tregs, which actually may differ from Teffs in their requirements for optimal activation, e.g., by different signal transduction moieties. Functional testing of bulk populations, e.g., for suppressive activity, or expansion are rather indirect, time consuming, and do not provide information on the level of individual cells. Therefore, we tested whether the Treg activation signature described here may allow fast functional *in vitro* testing of genetically engineered Tregs. To provide a controllable system for Treg activation, Treg specificity was redirected toward an innocuous exogenous antigen. To this end, a Dex-specific CAR construct was designed (Figure 4A) and CAR–Tregs were generated by lentiviral transduction. CAR–Tregs could be identified by surface expression of LNGFR (Figures 4B,C) and Dex was bound by CAR–Tregs, indicating functional receptor expression and antigen binding (Figure 4D). Following antigen-specific stimulation with soluble or bead-bound Dex, CD137 was upregulated selectively on LNGFR+ cells, but not on LNGFR− cells within the same culture (Figures 4E,F; Figures S1A,B in Supplementary Material). Next, the functionality of different extracellular spacer and intracellular signaling domains to activate Tregs *in vitro* was analyzed by CD137 expression. To this end, we generated different CAR constructs with long (L, 228aa), medium (M, 119aa), short (S, 45aa), and very short (XS, 12aa) extracellular spacer domains (Figures S2A,B in Supplementary Material). In spite of superior Dex binding by S spacers (Figure 5A), CD137 expression was only efficiently upregulated on CAR–Tregs with XS spacers even among cells that had bound Dex (Figure 5B). To investigate the impact of costimulation on CAR–Treg functionality, we generated several different Dex-specific CAR constructs with an optimized XS spacer consisting of CD3ζ signaling combined with CD28, CD137, ICOS, CD134, or PD-1 costimulation. To control for the effect of costimulation alone, we generated CAR constructs with CD28–CDε and CD137–CDε signaling (Figure S2C in Supplementary Material). In spite of similar transduction rates as determined by LNGFR expression (Figure S2D in Supplementary Material), CAR constructs differed in their ability to bind Dex (Figure S2E in Supplementary Material). Particularly CAR–Tregs with ICOS costimulation and CDε signaling exhibited impaired binding of Dex, indicating inefficient CAR surface expression that potentially derives from structural inhibitions that result in unstable CAR expression (59–61). Although the remaining constructs were similarly able to bind Dex, only CAR–Tregs containing CD137–CD3ζ or to a lesser extent CD134–CD3ζ signaling were activated, but not CAR–Tregs that contained, e.g., commonly used CD28 costimulation (Figure 5C). Next, the impact of costimulation on the expansion of CAR–Tregs was analyzed. To directly compare the CAR constructs within a single culture, CAR–Tregs with different signaling domains were pooled and expansion of the different constructs was determined by quantitative real-time PCR. There was an enrichment of LNGFR+ cells in the presence of Dex compared to stimulation with anti-CD3/anti-CD28 (Figure 5D). To determine selective expansion of a particular construct, primers spanning construct-specific regions within the intracellular signaling domain were designed and expression was calculated relative to the beginning of the culture. Within this competitive co-culture, there was a selective expansion of CAR–Tregs with CD137–CD3ζ signaling in the presence of Dex (Figure 5E), while polyclonal expansion did not favor any construct (Figure 5F). Interestingly, there was minor expansion with CD28 costimulation in some donors, while CD134 did not induce CAR–Treg proliferation. Taken together, CD137 expression enabled the rapid evaluation of various spacer and signaling domains for CAR-mediated Treg activation *in vitro* revealing the superiority of CD137–CD3ζ signaling over CD28 costimulation for CAR–Treg functionality.

### CD137+CD154− Expression Identifies Antigen-Activated FoxP3+ CAR–Tregs

**Figure 6:**
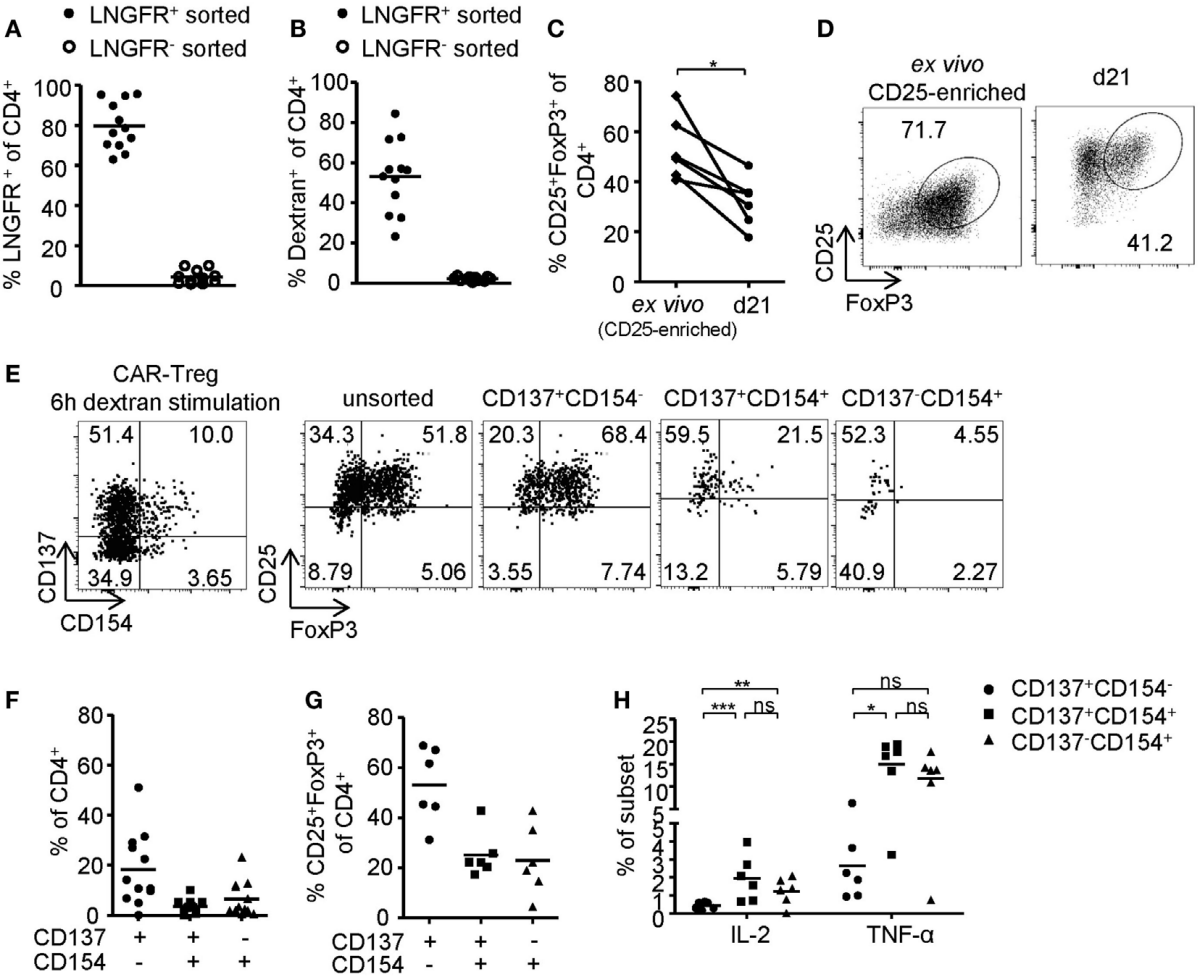
Phenotype of antigen-activated chimeric antigen receptor (CAR)–regulatory T cells (Tregs). **(A–H)** LNGFR+ CAR–Tregs were sorted and expanded for 10–12 days before analysis of **(A)** LNGFR expression and **(B)** dextran-binding (*n* = 12 for LNGFR+ sorted and *n* = 11 for LNGFR− sorted from four independent experiments). **(C,D)** FoxP3 expression in LNGFR+ sorted CAR–Tregs was analyzed *ex vivo* and after 21 days; **(D)** representative dot plot of one donor and **(C)** statistical summary of several donors (*n* = 6 from two independent experiments). **(E–H)** CAR–Tregs were restimulated for 6 h with bead-bound dextran and **(E,G)** FoxP3 expression (*n* = 6 from two independent experiments) and **(E,F)** CD137 and CD154 expression (*n* = 12 for from four independent experiments) were analyzed; **(E)** representative dot plot of one donor and **(F,G)** statistical summary of several donors. **(H)** Cytokine expression was analyzed on CD137- and CD154-expressing CAR–Tregs (*n* = 6 from two independent experiments). **(A–C,F–H)** Each dot represents one donor, and statistical significances were determined by **(C)** paired *t*-test or **(H)** Wilcoxon signed-rank test; lines indicate **(A,B)** median or **(F–H)** mean.

*In vitro* generated antigen-specific Tregs require increased safety measures to prevent contaminations with potentially autoaggressive Teffs. To this end, CAR–Tregs with an optimized extracellular XS spacer and intracellular CD137–CD3ζ signaling domain were generated and sorted by LNGFR expression. While transgene expression (Figure 6A) and dextran binding (Figure 6B) were maintained upon expansion, FoxP3 expression was significantly reduced compared to *ex vivo* isolated Tregs (Figures 6C,D). Following 6-h antigen-specific stimulation, CD137 and CD154 were upregulated by dextran-reactive cells revealing variable frequencies of CD154-expressing cells, indicating the presence of significant numbers of CAR-expressing non-Tregs within this culture (Figures 6E,F). In line with our observations after polyclonal stimulation (Figures 2A–D and 3B), antigen-specific CD137+CAR– Tregs that lacked CD154 expression were characterized by high levels of FoxP3 expression (Figures 6E,G), low levels of TNF-α, and complete absence of IL-2 expression (Figure 6H). In contrast, CD154 upregulation identified dextran-reactive cells that expressed low levels of FoxP3 and high levels of IL-2 and TNF-α (Figures 6G,H) and therefore represent antigen-specific non-Tregs with a significant inflammatory potential, which may cause adverse effects upon transfer *in vivo*. Taken together, we identified CD137+CD154− expression as Treg-specific activation signature, which enabled rapid analysis of *in vitro* generated Tregs in regard to their activation and stability emerging as a novel tool for the optimization of Treg efficacy and purity, e.g., for therapeutic applications.

## Discussion

The potential of *in vitro* generated Tregs to control chronic inflammatory diseases emerges as important target for clinical applications. To date, stability of expanded Tregs depends on the purity of the starting population as there are currently no unambiguous markers to separate stable Tregs from Teffs after expansion. In this study, we present a Treg-specific activation signature that enables the identification of epigenetically stable antigen-activated Tregs not only *ex vivo* but also following prolonged *in vitro* activation of human Tregs, which provides the opportunity to identify and purify Tregs after prior expansion.

*In vitro* generation of Tregs by large-scale expansion and/or genetic engineering remains a major challenge as there are currently no markers for the unambiguous identification of Tregs *ex vivo* or after prior *in vitro* culture. It has been proposed that naive Tregs represent a particularly stable Treg subset (62–66). Indeed, cord blood-derived CD25+ Tregs, consisting mainly of naive Tregs, were successfully expanded *in vitro* and have proven safety and efficacy in allogeneic umbilical cord blood transplantation (3–5). However, limited availability of cord blood and low frequencies of naive Tregs in adult blood currently prevent their large-scale clinical application. Alternatively, expanded FACS-sorted CD25+CD127− Tregs have been used in autologous settings for treatment of autoimmunity (8, 12). Yet, FACS sorting is still not routinely applicable under GMP conditions, and even expansion of FACS-sorted CD25+CD127− Tregs fails to eliminate non-Treg contaminations (67, 68). Clinical Treg isolation protocols are largely based on magnetic separation of CD25-expressing T cells (1, 4, 7, 69–71). Although this enriches FoxP3+ Tregs *ex vivo*, purity is highly variable and depends on the composition of the starting material, i.e., cord blood, adult peripheral blood, or leukapheresis. Furthermore, non-Tregs can acquire a CD25+CD127− phenotype *in vivo* or upon *in vitro* culture and upregulate FoxP3 without acquisition of regulatory functions (72–75). Therefore, the lack of markers for the isolation of Tregs *ex vivo* as well as for their purification after prior expansion represents a significant technological challenge. Furthermore, the functional optimization of Tregs is hampered by the lack of Treg-specific activation markers, which allow discrimination between activated Tregs and activated Teffs to permit a direct evaluation of signals and pathways required for Tregs *versus* Teffs. Thus, the Treg-specific activation signature identified here provides a convenient tool to improve purity and function of human Tregs and to overcome a major hurdle for *in vitro* generation of functionally optimized Tregs for therapeutic applications.

In this study, CD137+CD154− expression was shown to selectively identify epigenetically stable antigen-activated Tregs within *in vitro* expanded cultures. It has been shown that FoxP3 expression is unable to distinguish between Tregs and non-Tregs after prolonged *in vitro* expansion, whereas TSDR demethylation enabled unambiguous identification of suppressive Treg clones (76). We show a striking linear correlation of CD137+CD154− expression with TSDR demethylation revealing the potential of this surface marker combination to identify epigenetically stable FoxP3+ Tregs. Treg instability has been observed upon *in vitro* expansion resulting in the loss of FoxP3 expression and acquisition of effector functions (62–65, 77) and also notable numbers of IFN-γ-producing CD25+CD127− cells have been shown in patients suffering from MS (78), T1D (79), arthritis (80–82), psoriasis (83), or inflammatory bowel disease (84–86). Furthermore, studies from mice have shown that Treg plasticity contributes to anti-helminth immune responses (87), but also to heighten chronic inflammation (81, 88, 89) and allergy (90). Collectively, the contribution of Treg plasticity to immune responses and tolerance in humans remain to be determined in future studies, yet its potential to exaggerate immune pathologies represents a significant safety risk for adoptive transfer. Here, we show that plasticity including upregulation of effector functions and downregulation of FoxP3 as well as impaired suppressive capacities were restricted to CD154+ cells. With regard to their highly methylated TSDR, it can be assumed that CD137+CD154+ and CD137−CD154+ cells contain significant frequencies of contaminating effector cells. Since CD137+CD154+ Tregs contained higher levels of FoxP3+ Tregs compared to cells that lacked CD137 expression, it can be hypothesized that instable Tregs were enriched within this subset, while CD137+CD154− Tregs were of remarkable phenotypic stability, which was mediated by a robust epigenetic Treg signature.

Here, we show a notable correlation of CD137 expression with a Treg phenotype, including FoxP3 expression and epigenetic Treg identity. Stable FoxP3 expression is dependent on TSDR demethylation (42–45), and it has been shown that cooperative complexes of FoxP3 and NFAT can directly regulate gene expression by suppressing effector molecules (e.g., IL-2) while upregulating Treg-associated genes (e.g., CTLA4) (91). Furthermore, Marson et al. showed that *tnfrsf9* (CD137) is a direct target of FoxP3 (92) providing a possible link between CD137 expression with a stable Treg signature that was shown here. Yet, CD137 expression is not limited to the Treg lineage, but can also be upregulated by CD4+ Teffs upon prolonged stimulation (48, 93). To account for the different kinetics of CD137 expression on Tregs and Teffs, it can be speculated that accessibility of the region is regulated by epigenetic modifications as CD137 has been shown to be hypomethylated in Tregs compared to Teffs (58). Similarly, *tnfrsf9* was almost completely demethylated in CD137+CD154− Tregs while *cd40lg* (CD154) was highly methylated providing a molecular basis for rapid CD137 upregulation and lack of CD154 expression on stable Tregs. The importance of CD137 as costimulator for T cell activation has been well established (94), yet its role in Tregs remains elusive. CD137L is expressed on a variety of APCs and activated T cells and studies have shown that CD137–CD137L interaction increases Treg function (95–99) although impaired suppressive capacity has also been reported (100). Therefore, the function of CD137 expression on human Tregs remains to be determined, yet its rapid upregulation on Tregs could provide a versatile and wide-ranging mechanism enabling regulatory interactions with various immune cells (101).

Redirecting Treg specificity by CARs is currently emerging as a promising approach for increased efficacy of therapeutic Tregs (33–41). However, the generation of functional CAR–Tregs *in vivo* requires evaluation of their functionality *in vitro*, which is currently limited due to the lack of Treg-specific activation markers. LAP and GARP have been shown to be upregulated on Tregs after polyclonal (49, 51, 102, 103) or alloantigen-specific (50) stimulation, yet expression requires prolonged stimulation and does not necessarily correlate with FoxP3 expression (104–106). Here, CD137 was shown to be upregulated selectively on FoxP3+ Tregs after 6-h stimulation, which enabled the rapid identification, isolation, and characterization of antigen-activated Tregs. To generate a controllable system for Treg activation, we redirected Treg specificity toward dextran as a model antigen. Dextran naturally exists in different sizes depending on the molecular weight and can be applied in different forms (e.g., soluble vs. bead-bound) providing a system for the rapid analysis of the effect of antigen binding on Treg activation. To date, little is known about the requirements of Treg activation *via* CAR stimulation, and individual CARs may differ in their specific requirements to optimally activate T cells (107). It has been proposed that the extracellular spacer (53, 108–112) and the intracellular signaling domain (113–119) have a significant impact on the functionality of CAR–T cells. Activation-induced CD137 expression enabled the rapid comparison of different CAR constructs in their ability to activate Tregs revealing superiority of CD137 costimulation compared to commonly used CD28–CD3ζ signaling. It has been shown that CD28, but not CD137 costimulation, can initiate tonic signaling in conventional T cells leading to an exhaustion phenotype and limited efficacy (107). However, in that particular study, the degree of exhaustion varied between CARs with different scFv domains in spite of identical signaling domains. Therefore, it can be speculated that CD28 costimulation can be a potent inducer of T cell activation depending on the CAR. Nevertheless, it was shown here that CD137–CD3ζ signaling was superior for *in vitro* CAR–Treg activation and expansion indicating potentially different signaling requirements than Tcon.

In conclusion, we show CD137+CD154− expression to be a highly specific Treg activation signature that enabled the identification and isolation of stable Tregs even after prolonged *in vitro* culture. We also show that this short-term activation signature allowed rapid screening and optimization of CAR functionality in Tregs. Taken together, universal application of this Treg-specific activation signature will greatly improve Treg selection and functional optimization, such as for clinical applications in Treg-based therapies.

## Ethics Statement

Peripheral blood was obtained from healthy donors that gave informed consent in accordance with the recommendations of the local ethics committee of the Charité Berlin. All subjects gave written informed consent in accordance with the Declaration of Helsinki. The protocol was approved by the local ethics committee of the Charité Berlin.

## Author Contributions

AN designed and performed experiments, interpreted the data, and wrote the manuscript. DL and AK generated CAR constructs and primers. PB designed experiments and interpreted the data. TH performed experiments. KV and BS performed TSDR analysis. JG, PG, JP, and JW designed, performed, and interpreted data from deep bisulfite amplicon sequencing. AS designed this study, interpreted the data, and wrote the manuscript. All authors provided discussion and reviewed the manuscript.

## Conflict of Interest Statement

AK and DL are employees of Miltenyi Biotec. AS is a consultant for Miltenyi Biotec. All other authors declare that the research was conducted in the absence of any commercial or financial relationships that could be construed as a potential conflict of interest.

## Abbreviations

Treg, regulatory T cell; Teff, effector T cell; CAR, chimeric antigen receptor; TCR, T cell receptor; scFv, single-chain variable fragment; Dex, dextran; GvHD, graft-versus-host disease; T1D, type 1 diabetes; EAE, experimental autoimmune encephalomyelitis; TSDR, Treg-specific demethylated region.

## References

[1] Di Ianni M Falzetti F Carotti A Terenzi A Castellino F Bonifacio E Tregs prevent GVHD and promote immune reconstitution in HLA-haploidentical transplantation. Blood (2011) 117:3921–8. 10.1182/blood-2010-10-311894 21292771

[2] Martelli MF Di Ianni M Ruggeri L Falzetti F Carotti A Terenzi A HLA-haploidentical transplantation with regulatory and conventional T-cell adoptive immunotherapy prevents acute leukemia relapse. Blood (2014) 124:638–44. 10.1182/blood-2014-03-564401 24923299

[3] Brunstein CG Blazar BR Miller JS Cao Q Hippen KL McKenna DH Adoptive transfer of umbilical cord blood-derived regulatory T cells and early viral reactivation. Biol Blood Marrow Transplant (2013) 19:1271–3. 10.1016/j.bbmt.2013.06.004 PMC3870093 23806771

[4] Brunstein CG Miller JS Cao Q McKenna DH Hippen KL Curtsinger J Infusion of ex vivo expanded T regulatory cells in adults transplanted with umbilical cord blood: safety profile and detection kinetics. Blood (2011) 117:1061–70. 10.1182/blood-2010-07-293795 20952687 PMC3035067

[5] Brunstein CG Miller JS McKenna DH Hippen KL DeFor TE Sumstad D Umbilical cord blood-derived T regulatory cells to prevent GVHD: kinetics, toxicity profile, and clinical effect. Blood (2016) 127:1044–51. 10.1182/blood-2015-06-653667 26563133 PMC4768428

[6] Edinger M Hoffmann P. Regulatory T cells in stem cell transplantation: strategies and first clinical experiences. Curr Opin Immunol (2011) 23:679–84. 10.1016/j.coi.2011.06.006 21802270

[7] Theil A Tuve S Oelschlägel U Maiwald A Döhler D Oßmann D Adoptive transfer of allogeneic regulatory T cells into patients with chronic graft-versus-host disease. Cytotherapy (2015) 17:473–86. 10.1016/j.jcyt.2014.11.005 25573333

[8] Trzonkowski P Bieniaszewska M Juścińska J Dobyszuk A Krzystyniak A Marek N First-in-man clinical results of the treatment of patients with graft versus host disease with human ex vivo expanded CD4+CD25+CD127- T regulatory cells. Clin Immunol (2009) 133:22–6. 10.1016/j.clim.2009.06.001 19559653

[9] Bluestone JA Buckner JH Fitch M Gitelman SE Gupta S Hellerstein MK Type 1 diabetes immunotherapy using polyclonal regulatory T cells. Sci Transl Med (2015) 7:315ra189. 10.1126/scitranslmed.aad4134 26606968 PMC4729454

[10] Marek-Trzonkowska N Myśliwec M Siebert J Trzonkowski P. Clinical application of regulatory T cells in type 1 diabetes. Pediatr Diabetes (2013) 14:322–32. 10.1111/pedi.12029 23627860

[11] Marek-Trzonkowska N Myśliwiec M Dobyszuk A Grabowska M Derkowska I Juścińska J Therapy of type 1 diabetes with CD4(+)CD25(high)CD127-regulatory T cells prolongs survival of pancreatic islets – results of one year follow-up. Clin Immunol (2014) 153:23–30. 10.1016/j.clim.2014.03.016 24704576

[12] Marek-Trzonkowska N Mysliwiec M Dobyszuk A Grabowska M Techmanska I Juscinska J Administration of CD4+CD25highCD127- regulatory T cells preserves beta-cell function in type 1 diabetes in children. Diabetes Care (2012) 35:1817–20. 10.2337/dc12-0038 22723342 PMC3425004

[13] Marek-Trzonkowska N Myśliwiec M Iwaszkiewicz-Grześ D Gliwiński M Derkowska I Żalińska M Factors affecting long-term efficacy of T regulatory cell-based therapy in type 1 diabetes. J Transl Med (2016) 14:332. 10.1186/s12967-016-1090-7 27903296 PMC5131539

[14] Masteller EL Warner MR Tang Q Tarbell KV McDevitt H Bluestone JA. Expansion of functional endogenous antigen-specific CD4+CD25+ regulatory T cells from nonobese diabetic mice. J Immunol (2005) 175:3053–9. 10.4049/jimmunol.175.5.3053 16116193

[15] Tang Q Henriksen KJ Bi M Finger EB Szot G Ye J In vitro-expanded antigen-specific regulatory T cells suppress autoimmune diabetes. J Exp Med (2004) 199:1455–65. 10.1084/jem.20040139 15184499 PMC2211775

[16] Tarbell KV Petit L Zuo X Toy P Luo X Mqadmi A Dendritic cell-expanded, islet-specific CD4+ CD25+ CD62L+ regulatory T cells restore normoglycemia in diabetic NOD mice. J Exp Med (2007) 204:191–201. 10.1084/jem.20061631 17210729 PMC2118426

[17] Tarbell KV Yamazaki S Olson K Toy P Steinman RM. CD25+ CD4+ T cells, expanded with dendritic cells presenting a single autoantigenic peptide, suppress autoimmune diabetes. J Exp Med (2004) 199:1467–77. 10.1084/jem.20040180 15184500 PMC2211787

[18] Golshayan D Jiang S Tsang J Garin MI Mottet C Lechler RI. In vitro-expanded donor alloantigen-specific CD4+CD25+ regulatory T cells promote experimental transplantation tolerance. Blood (2007) 109:827–35. 10.1182/blood-2006-05-025460 17003369

[19] Joffre O Santolaria T Calise D Al Saati T Hudrisier D Romagnoli P Prevention of acute and chronic allograft rejection with CD4+CD25+Foxp3+ regulatory T lymphocytes. Nat Med (2008) 14:88–92. 10.1038/nm1688 18066074 PMC2443705

[20] Nishimura E Sakihama T Setoguchi R Tanaka K Sakaguchi S. Induction of antigen-specific immunologic tolerance by in vivo and in vitro antigen-specific expansion of naturally arising Foxp3+CD25+CD4+ regulatory T cells. Int Immunol (2004) 16:1189–201. 10.1093/intimm/dxh122 15237110

[21] Sagoo P Ali N Garg G Nestle FO Lechler RI Lombardi G. Human regulatory T cells with alloantigen specificity are more potent inhibitors of alloimmune skin graft damage than polyclonal regulatory T cells. Sci Transl Med (2011) 3:83ra42. 10.1126/scitranslmed.3002076 21593402 PMC3776382

[22] Tsang JY Ratnasothy K Li D Chen Y Bucy RP Lau KF The potency of allospecific Tregs cells appears to correlate with T cell receptor functional avidity. Am J Transplant (2011) 11:1610–20. 10.1111/j.1600-6143.2011.03650.x 21797973

[23] Tsang JY Tanriver Y Jiang S Xue SA Ratnasothy K Chen D Conferring indirect allospecificity on CD4+CD25+ Tregs by TCR gene transfer favors transplantation tolerance in mice. J Clin Invest (2008) 118:3619–28. 10.1172/JCI33185 18846251 PMC2564608

[24] Trenado A Charlotte F Fisson S Yagello M Klatzmann D Salomon BL Recipient-type specific CD4+CD25+ regulatory T cells favor immune reconstitution and control graft-versus-host disease while maintaining graft-versus-leukemia. J Clin Invest (2003) 112:1688–96. 10.1172/JCI17702 14660744 PMC281639

[25] Putnam AL Safinia N Medvec A Laszkowska M Wray M Mintz MA Clinical grade manufacturing of human alloantigen-reactive regulatory T cells for use in transplantation. Am J Transplant (2013) 13:3010–20. 10.1111/ajt.12433 24102808 PMC4161737

[26] Stephens LA Malpass KH Anderton SM. Curing CNS autoimmune disease with myelin-reactive Foxp3+ Treg. Eur J Immunol (2009) 39:1108–17. 10.1002/eji.200839073 19350586

[27] Kieback E Hilgenberg E Stervbo U Lampropoulou V Shen P Bunse M Thymus-derived regulatory T cells are positively selected on natural self-antigen through cognate interactions of high functional avidity. Immunity (2016) 44:1114–26. 10.1016/j.immuni.2016.04.018 27192577

[28] Wright GP Notley CA Xue SA Bendle GM Holler A Schumacher TN Adoptive therapy with redirected primary regulatory T cells results in antigen-specific suppression of arthritis. Proc Natl Acad Sci U S A (2009) 106:19078–83. 10.1073/pnas.0907396106 19884493 PMC2776462

[29] Fujio K Okamoto A Araki Y Shoda H Tahara H Tsuno NH Gene therapy of arthritis with TCR isolated from the inflamed paw. J Immunol (2006) 177:8140–7. 10.4049/jimmunol.177.11.8140 17114489

[30] Brusko TM Koya RC Zhu S Lee MR Putnam AL McClymont SA Human antigen-specific regulatory T cells generated by T cell receptor gene transfer. PLoS One (2010) 5:e11726. 10.1371/journal.pone.0011726 20668510 PMC2908680

[31] Plesa G Zheng L Medvec A Wilson CB Robles-Oteiza C Liddy N TCR affinity and specificity requirements for human regulatory T-cell function. Blood (2012) 119:3420–30. 10.1182/blood-2011-09-377051 22318202 PMC3325034

[32] Hull CM Nickolay LE Estorninho M Richardson MW Riley JL Peakman M Generation of human islet-specific regulatory T cells by TCR gene transfer. J Autoimmun (2017) 79:63–73. 10.1016/j.jaut.2017.01.001 28117148

[33] Mekala DJ Geiger TL. Immunotherapy of autoimmune encephalomyelitis with redirected CD4+CD25+ T lymphocytes. Blood (2005) 105:2090–2. 10.1182/blood-2004-09-3579 15528313

[34] Blat D Zigmond E Alteber Z Waks T Eshhar Z. Suppression of murine colitis and its associated cancer by carcinoembryonic antigen-specific regulatory T cells. Mol Ther (2014) 22:1018–28. 10.1038/mt.2014.41 24686242 PMC4015241

[35] Elinav E Adam N Waks T Eshhar Z. Amelioration of colitis by genetically engineered murine regulatory T cells redirected by antigen-specific chimeric receptor. Gastroenterology (2009) 136:1721–31. 10.1053/j.gastro.2009.01.049 19208357

[36] Elinav E Waks T Eshhar Z. Redirection of regulatory T cells with predetermined specificity for the treatment of experimental colitis in mice. Gastroenterology (2008) 134:2014–24. 10.1053/j.gastro.2008.02.060 18424268

[37] Boardman DA Philippeos C Fruhwirth GO Ibrahim MA Hannen RF Cooper D Expression of a chimeric antigen receptor specific for donor HLA class I enhances the potency of human regulatory T cells in preventing human skin transplant rejection. Am J Transplant (2017) 17:931–43. 10.1111/ajt.14185 28027623

[38] MacDonald KG Hoeppli RE Huang Q Gillies J Luciani DS Orban PC Alloantigen-specific regulatory T cells generated with a chimeric antigen receptor. J Clin Invest (2016) 126:1413–24. 10.1172/JCI82771 26999600 PMC4811124

[39] Noyan F Zimmermann K Hardtke-Wolenski M Knoefel A Schulde E Geffers R Prevention of allograft rejection by use of regulatory T cells with an MHC-specific chimeric antigen receptor. Am J Transplant (2017) 17:917–30. 10.1111/ajt.14175 27997080

[40] Skuljec J Chmielewski M Happle C Habener A Busse M Abken H Chimeric antigen receptor-redirected regulatory T cells suppress experimental allergic airway inflammation, a model of asthma. Front Immunol (2017) 8:1125. 10.3389/fimmu.2017.01125 28955341 PMC5600908

[41] Yoon J Schmidt A Zhang AH Königs C Kim YC Scott DW. FVIII-specific human chimeric antigen receptor T-regulatory cells suppress T- and B-cell responses to FVIII. Blood (2017) 129:238–45. 10.1182/blood-2016-07-727834 28064157 PMC5234219

[42] Baron U Floess S Wieczorek G Baumann K Grützkau A Dong J DNA demethylation in the human FOXP3 locus discriminates regulatory T cells from activated FOXP3(+) conventional T cells. Eur J Immunol (2007) 37:2378–89. 10.1002/eji.200737594 17694575

[43] Floess S Freyer J Siewert C Baron U Olek S Polansky J Epigenetic control of the foxp3 locus in regulatory T cells. PLoS Biol (2007) 5:e38. 10.1371/journal.pbio.0050038 17298177 PMC1783672

[44] Huehn J Polansky JK Hamann A. Epigenetic control of FOXP3 expression: the key to a stable regulatory T-cell lineage? Nat Rev Immunol (2009) 9:83–9. 10.1038/nri2474 19114986

[45] Polansky JK Kretschmer K Freyer J Floess S Garbe A Baron U DNA methylation controls Foxp3 gene expression. Eur J Immunol (2008) 38:1654–63. 10.1002/eji.200838105 18493985

[46] Bacher P Heinrich F Stervbo U Nienen M Vahldieck M Iwert C Regulatory T cell specificity directs tolerance versus allergy against aeroantigens in humans. Cell (2016) 167:1067.e–78.e. 10.1016/j.cell.2016.09.050 27773482

[47] Bacher P Kniemeyer O Schönbrunn A Sawitzki B Assenmacher M Rietschel E Antigen-specific expansion of human regulatory T cells as a major tolerance mechanism against mucosal fungi. Mucosal Immunol (2014) 7:916–28. 10.1038/mi.2013.107 24301658

[48] Schoenbrunn A Frentsch M Kohler S Keye J Dooms H Moewes B A converse 4-1BB and CD40 ligand expression pattern delineates activated regulatory T cells (Treg) and conventional T cells enabling direct isolation of alloantigen-reactive natural Foxp3+ Treg. J Immunol (2012) 189:5985–94. 10.4049/jimmunol.1201090 23162126

[49] Tran DQ Andersson J Wang R Ramsey H Unutmaz D Shevach EM. GARP (LRRC32) is essential for the surface expression of latent TGF-beta on platelets and activated FOXP3+ regulatory T cells. Proc Natl Acad Sci U S A (2009) 106:13445–50. 10.1073/pnas.0901944106 19651619 PMC2726354

[50] Noyan F Lee YS Zimmermann K Hardtke-Wolenski M Taubert R Warnecke G Isolation of human antigen-specific regulatory T cells with high suppressive function. Eur J Immunol (2014) 44:2592–602. 10.1002/eji.201344381 24990119

[51] Tran DQ Andersson J Hardwick D Bebris L Illei GG Shevach EM. Selective expression of latency-associated peptide (LAP) and IL-1 receptor type I/II (CD121a/CD121b) on activated human FOXP3+ regulatory T cells allows for their purification from expansion cultures. Blood (2009) 113:5125–33. 10.1182/blood-2009-01-199950 19299332 PMC2686183

[52] Seddiki N Cook L Hsu DC Phetsouphanh C Brown K Xu Y Human antigen-specific CD4(+) CD25(+) CD134(+) CD39(+) T cells are enriched for regulatory T cells and comprise a substantial proportion of recall responses. Eur J Immunol (2014) 44:1644–61. 10.1002/eji.201344102 24752698

[53] Hudecek M Sommermeyer D Kosasih PL Silva-Benedict A Liu L Rader C The nonsignaling extracellular spacer domain of chimeric antigen receptors is decisive for in vivo antitumor activity. Cancer Immunol Res (2015) 3:125–35. 10.1158/2326-6066.CIR-14-0127 25212991 PMC4692801

[54] Lutsik P Feuerbach L Arand J Lengauer T Walter J Bock C. BiQ Analyzer HT: locus-specific analysis of DNA methylation by high-throughput bisulfite sequencing. Nucleic Acids Res (2011) 39:W551–6. 10.1093/nar/gkr312 21565797 PMC3125748

[55] Durek P Nordström K Gasparoni G Salhab A Kressler C de Almeida M Epigenomic profiling of human CD4+ T cells supports a linear differentiation model and highlights molecular regulators of memory development. Immunity (2016) 45:1148–61. 10.1016/j.immuni.2016.10.022 27851915

[56] Bacher P Kniemeyer O Teutschbein J Thön M Vödisch M Wartenberg D Identification of immunogenic antigens from Aspergillus fumigatus by direct multiparameter characterization of specific conventional and regulatory CD4+ T cells. J Immunol (2014) 193:3332–43. 10.4049/jimmunol.1400776 25172488

[57] Arvey A van der Veeken J Plitas G Rich SS Concannon P Rudensky AY. Genetic and epigenetic variation in the lineage specification of regulatory T cells. Elife (2015) 4:e07571. 10.7554/eLife.07571 26510014 PMC4623597

[58] Schmidl C Klug M Boeld TJ Andreesen R Hoffmann P Edinger M Lineage-specific DNA methylation in T cells correlates with histone methylation and enhancer activity. Genome Res (2009) 19:1165–74. 10.1101/gr.091470.109 19494038 PMC2704427

[59] Heuser C Hombach A Lösch C Manista K Abken H. T-cell activation by recombinant immunoreceptors: impact of the intracellular signalling domain on the stability of receptor expression and antigen-specific activation of grafted T cells. Gene Ther (2003) 10:1408–19. 10.1038/sj.gt.3302023 12900755

[60] Nolan KF Yun CO Akamatsu Y Murphy JC Leung SO Beecham EJ Bypassing immunization: optimized design of “designer T cells” against carcinoembryonic antigen (CEA)-expressing tumors, and lack of suppression by soluble CEA. Clin Cancer Res (1999) 5:3928–41. 10632322

[61] Zhao Y Wang QJ Yang S Kochenderfer JN Zheng Z Zhong X A herceptin-based chimeric antigen receptor with modified signaling domains leads to enhanced survival of transduced T lymphocytes and antitumor activity. J Immunol (2009) 183:5563–74. 10.4049/jimmunol.0900447 19843940 PMC6292203

[62] d’Hennezel E Yurchenko E Sgouroudis E Hay V Piccirillo CA. Single-cell analysis of the human T regulatory population uncovers functional heterogeneity and instability within FOXP3+ cells. J Immunol (2011) 186:6788–97. 10.4049/jimmunol.1100269 21576508

[63] Hoffmann P Boeld TJ Eder R Huehn J Floess S Wieczorek G Loss of FOXP3 expression in natural human CD4+CD25+ regulatory T cells upon repetitive in vitro stimulation. Eur J Immunol (2009) 39:1088–97. 10.1002/eji.200838904 19283780

[64] Hoffmann P Eder R Boeld TJ Doser K Piseshka B Andreesen R Only the CD45RA+ subpopulation of CD4+CD25high T cells gives rise to homogeneous regulatory T-cell lines upon in vitro expansion. Blood (2006) 108:4260–7. 10.1182/blood-2006-06-027409 16917003

[65] Arroyo Hornero R Betts GJ Sawitzki B Vogt K Harden PN Wood KJ. CD45RA distinguishes CD4+CD25+CD127-/low TSDR demethylated regulatory T cell subpopulations with differential stability and susceptibility to tacrolimus-mediated inhibition of suppression. Transplantation (2017) 101:302–9. 10.1097/TP.0000000000001278 28118317 PMC5265687

[66] Miyara M Yoshioka Y Kitoh A Shima T Wing K Niwa A Functional delineation and differentiation dynamics of human CD4+ T cells expressing the FoxP3 transcription factor. Immunity (2009) 30:899–911. 10.1016/j.immuni.2009.03.019 19464196

[67] Putnam AL Brusko TM Lee MR Liu W Szot GL Ghosh T Expansion of human regulatory T-cells from patients with type 1 diabetes. Diabetes (2009) 58:652–62. 10.2337/db08-1168 19074986 PMC2646064

[68] Seay HR Putnam AL Cserny J Posgai AL Rosenau EH Wingard JR Expansion of human Tregs from cryopreserved umbilical cord blood for GMP-compliant autologous adoptive cell transfer therapy. Mol Ther Methods Clin Dev (2017) 4:178–91. 10.1016/j.omtm.2016.12.003 28345003 PMC5363324

[69] Ukena SN Höpting M Velaga S Ivanyi P Grosse J Baron U Isolation strategies of regulatory T cells for clinical trials: phenotype, function, stability, and expansion capacity. Exp Hematol (2011) 39:1152–60. 10.1016/j.exphem.2011.08.010 21864487

[70] Hippen KL Riley JL June CH Blazar BR. Clinical perspectives for regulatory T cells in transplantation tolerance. Semin Immunol (2011) 23:462–8. 10.1016/j.smim.2011.07.008 21820917 PMC3230779

[71] Hoffmann P Boeld TJ Eder R Albrecht J Doser K Piseshka B Isolation of CD4+CD25+ regulatory T cells for clinical trials. Biol Blood Marrow Transplant (2006) 12:267–74. 10.1016/j.bbmt.2006.01.005 16503495

[72] Allan SE Crome SQ Crellin NK Passerini L Steiner TS Bacchetta R Activation-induced FOXP3 in human T effector cells does not suppress proliferation or cytokine production. Int Immunol (2007) 19:345–54. 10.1093/intimm/dxm014 17329235

[73] Gavin MA Torgerson TR Houston E DeRoos P Ho WY Stray-Pedersen A Single-cell analysis of normal and FOXP3-mutant human T cells: FOXP3 expression without regulatory T cell development. Proc Natl Acad Sci U S A (2006) 103:6659–64. 10.1073/pnas.0509484103 16617117 PMC1458937

[74] Pillai V Ortega SB Wang CK Karandikar NJ. Transient regulatory T-cells: a state attained by all activated human T-cells. Clin Immunol (2007) 123:18–29. 10.1016/j.clim.2006.10.014 17185041 PMC1868523

[75] Wang J Ioan-Facsinay A van der Voort EI Huizinga TW Toes RE. Transient expression of FOXP3 in human activated nonregulatory CD4+ T cells. Eur J Immunol (2007) 37:129–38. 10.1002/eji.200636435 17154262

[76] Stockis J Fink W François V Connerotte T de Smet C Knoops L Comparison of stable human Treg and Th clones by transcriptional profiling. Eur J Immunol (2009) 39:869–82. 10.1002/eji.200838807 19224638

[77] Koenen HJ Smeets RL Vink PM van Rijssen E Boots AM Joosten I. Human CD25highFoxp3pos regulatory T cells differentiate into IL-17-producing cells. Blood (2008) 112:2340–52. 10.1182/blood-2008-01-133967 18617638

[78] Dominguez-Villar M Baecher-Allan CM Hafler DA. Identification of T helper type 1-like, Foxp3+ regulatory T cells in human autoimmune disease. Nat Med (2011) 17:673–5. 10.1038/nm.2389 21540856 PMC3675886

[79] McClymont SA Putnam AL Lee MR Esensten JH Liu W Hulme MA Plasticity of human regulatory T cells in healthy subjects and patients with type 1 diabetes. J Immunol (2011) 186:3918–26. 10.4049/jimmunol.1003099 21368230 PMC3091943

[80] Afzali B Mitchell PJ Edozie FC Povoleri GA Dowson SE Demandt L CD161 expression characterizes a subpopulation of human regulatory T cells that produces IL-17 in a STAT3-dependent manner. Eur J Immunol (2013) 43:2043–54. 10.1002/eji.201243296 23677517 PMC3815561

[81] Komatsu N Okamoto K Sawa S Nakashima T Oh-hora M Kodama T Pathogenic conversion of Foxp3+ T cells into TH17 cells in autoimmune arthritis. Nat Med (2014) 20:62–8. 10.1038/nm.3432 24362934

[82] Pesenacker AM Bending D Ursu S Wu Q Nistala K Wedderburn LR. CD161 defines the subset of FoxP3+ T cells capable of producing proinflammatory cytokines. Blood (2013) 121:2647–58. 10.1182/blood-2012-08-443473 23355538 PMC3617631

[83] Bovenschen HJ van de Kerkhof PC van Erp PE Woestenenk R Joosten I Koenen HJ. Foxp3+ regulatory T cells of psoriasis patients easily differentiate into IL-17A-producing cells and are found in lesional skin. J Invest Dermatol (2011) 131:1853–60. 10.1038/jid.2011.139 21654831

[84] Hovhannisyan Z Treatman J Littman DR Mayer L. Characterization of interleukin-17-producing regulatory T cells in inflamed intestinal mucosa from patients with inflammatory bowel diseases. Gastroenterology (2011) 140:957–65. 10.1053/j.gastro.2010.12.002 21147109 PMC3049831

[85] Kryczek I Wu K Zhao E Wei S Vatan L Szeliga W IL-17+ regulatory T cells in the microenvironments of chronic inflammation and cancer. J Immunol (2011) 186:4388–95. 10.4049/jimmunol.1003251 21357259

[86] Ueno A Jijon H Chan R Ford K Hirota C Kaplan GG Increased prevalence of circulating novel IL-17 secreting Foxp3 expressing CD4+ T cells and defective suppressive function of circulating Foxp3+ regulatory cells support plasticity between Th17 and regulatory T cells in inflammatory bowel disease patients. Inflamm Bowel Dis (2013) 19:2522–34. 10.1097/MIB.0b013e3182a85709 24097227

[87] Pelly VS Coomes SM Kannan Y Gialitakis M Entwistle LJ Perez-Lloret J Interleukin 4 promotes the development of ex-Foxp3 Th2 cells during immunity to intestinal helminths. J Exp Med (2017) 214(6):1809–26. 10.1084/jem.20161104 28507062 PMC5460998

[88] Bailey-Bucktrout SL Martinez-Llordella M Zhou X Anthony B Rosenthal W Luche H Self-antigen-driven activation induces instability of regulatory T cells during an inflammatory autoimmune response. Immunity (2013) 39:949–62. 10.1016/j.immuni.2013.10.016 24238343 PMC3912996

[89] Zhou X Bailey-Bucktrout SL Jeker LT Penaranda C Martínez-Llordella M Ashby M Instability of the transcription factor Foxp3 leads to the generation of pathogenic memory T cells in vivo. Nat Immunol (2009) 10:1000–7. 10.1038/ni.1774 19633673 PMC2729804

[90] Noval Rivas M Burton OT Wise P Charbonnier LM Georgiev P Oettgen HC Regulatory T cell reprogramming toward a Th2-cell-like lineage impairs oral tolerance and promotes food allergy. Immunity (2015) 42:512–23. 10.1016/j.immuni.2015.02.004 25769611 PMC4366316

[91] Wu Y Borde M Heissmeyer V Feuerer M Lapan AD Stroud JC FOXP3 controls regulatory T cell function through cooperation with NFAT. Cell (2006) 126:375–87. 10.1016/j.cell.2006.05.042 16873067

[92] Marson A Kretschmer K Frampton GM Jacobsen ES Polansky JK MacIsaac KD Foxp3 occupancy and regulation of key target genes during T-cell stimulation. Nature (2007) 445:931–5. 10.1038/nature05478 17237765 PMC3008159

[93] Wehler TC Karg M Distler E Konur A Nonn M Meyer RG Rapid identification and sorting of viable virus-reactive CD4(+) and CD8(+) T cells based on antigen-triggered CD137 expression. J Immunol Methods (2008) 339:23–37. 10.1016/j.jim.2008.07.017 18760281

[94] DeBenedette MA Chu NR Pollok KE Hurtado J Wade WF Kwon BS Role of 4-1BB ligand in costimulation of T lymphocyte growth and its upregulation on M12 B lymphomas by cAMP. J Exp Med (1995) 181:985–92. 10.1084/jem.181.3.985 7532686 PMC2191935

[95] Zheng G Wang B Chen A. The 4-1BB costimulation augments the proliferation of CD4+CD25+ regulatory T cells. J Immunol (2004) 173:2428–34. 10.4049/jimmunol.173.4.2428 15294956

[96] Zhang P Gao F Wang Q Wang X Zhu F Ma C Agonistic anti-4-1BB antibody promotes the expansion of natural regulatory T cells while maintaining Foxp3 expression. Scand J Immunol (2007) 66:435–40. 10.1111/j.1365-3083.2007.01994.x 17850588

[97] Elpek KG Yolcu ES Franke DD Lacelle C Schabowsky RH Shirwan H. Ex vivo expansion of CD4+CD25+FoxP3+ T regulatory cells based on synergy between IL-2 and 4-1BB signaling. J Immunol (2007) 179:7295–304. 10.4049/jimmunol.179.11.7295 18025172

[98] Kim J Kim W Kim HJ Park S Kim HA Jung D Host CD25+CD4+Foxp3+ regulatory T cells primed by anti-CD137 mAbs inhibit graft-versus-host disease. Biol Blood Marrow Transplant (2012) 18:44–54. 10.1016/j.bbmt.2011.09.004 21958951

[99] Lee J Lee EN Kim EY Park HJ Chang CY Jung DY Administration of agonistic anti-4-1BB monoclonal antibody leads to the amelioration of inflammatory bowel disease. Immunol Lett (2005) 101:210–6. 10.1016/j.imlet.2005.06.001 16026855

[100] Choi BK Bae JS Choi EM Kang WJ Sakaguchi S Vinay DS 4-1BB-dependent inhibition of immunosuppression by activated CD4+CD25+ T cells. J Leukoc Biol (2004) 75:785–91. 10.1189/jlb.1003491 14694186

[101] Kwon B. Is CD137 ligand (CD137L) signaling a fine tuner of immune responses? Immune Netw (2015) 15:121–4. 10.4110/in.2015.15.3.121 26140043 PMC4486774

[102] Stockis J Colau D Coulie PG Lucas S. Membrane protein GARP is a receptor for latent TGF-beta on the surface of activated human Treg. Eur J Immunol (2009) 39:3315–22. 10.1002/eji.200939684 19750484

[103] Wang R Kozhaya L Mercer F Khaitan A Fujii H Unutmaz D. Expression of GARP selectively identifies activated human FOXP3+ regulatory T cells. Proc Natl Acad Sci U S A (2009) 106:13439–44. 10.1073/pnas.0901965106 19666573 PMC2726405

[104] Abd Al Samid M Chaudhary B Khaled YS Ammori BJ Elkord E. Combining FoxP3 and Helios with GARP/LAP markers can identify expanded Treg subsets in cancer patients. Oncotarget (2016) 7:14083–94. 10.18632/oncotarget.7334 26885615 PMC4924699

[105] Gandhi R Farez MF Wang Y Kozoriz D Quintana FJ Weiner HL. Cutting edge: human latency-associated peptide+ T cells: a novel regulatory T cell subset. J Immunol (2010) 184:4620–4. 10.4049/jimmunol.0903329 20368276 PMC2904991

[106] Elkord E Abd Al Samid M Chaudhary B. Helios, and not FoxP3, is the marker of activated Tregs expressing GARP/LAP. Oncotarget (2015) 6:20026–36. 10.18632/oncotarget.4771 26343373 PMC4652984

[107] Long AH Haso WM Shern JF Wanhainen KM Murgai M Ingaramo M 4-1BB costimulation ameliorates T cell exhaustion induced by tonic signaling of chimeric antigen receptors. Nat Med (2015) 21:581–90. 10.1038/nm.3838 25939063 PMC4458184

[108] Guest RD Hawkins RE Kirillova N Cheadle EJ Arnold J O’Neill A The role of extracellular spacer regions in the optimal design of chimeric immune receptors: evaluation of four different scFvs and antigens. J Immunother (2005) 28:203–11. 10.1097/01.cji.0000161397.96582.59 15838376

[109] Hudecek M Lupo-Stanghellini MT Kosasih PL Sommermeyer D Jensen MC Rader C Receptor affinity and extracellular domain modifications affect tumor recognition by ROR1-specific chimeric antigen receptor T cells. Clin Cancer Res (2013) 19:3153–64. 10.1158/1078-0432.CCR-13-0330 23620405 PMC3804130

[110] James SE Greenberg PD Jensen MC Lin Y Wang J Till BG Antigen sensitivity of CD22-specific chimeric TCR is modulated by target epitope distance from the cell membrane. J Immunol (2008) 180:7028–38. 10.4049/jimmunol.180.10.7028 18453625 PMC2585549

[111] Hombach A Heuser C Gerken M Fischer B Lewalter K Diehl V T cell activation by recombinant FcepsilonRI gamma-chain immune receptors: an extracellular spacer domain impairs antigen-dependent T cell activation but not antigen recognition. Gene Ther (2000) 7:1067–75. 10.1038/sj.gt.3301195 10871757

[112] Wilkie S Picco G Foster J Davies DM Julien S Cooper L Retargeting of human T cells to tumor-associated MUC1: the evolution of a chimeric antigen receptor. J Immunol (2008) 180:4901–9. 10.4049/jimmunol.180.7.4901 18354214

[113] Finney HM Akbar AN Lawson AD. Activation of resting human primary T cells with chimeric receptors: costimulation from CD28, inducible costimulator, CD134, and CD137 in series with signals from the TCR zeta chain. J Immunol (2004) 172:104–13. 10.4049/jimmunol.172.1.104 14688315

[114] Kowolik CM Topp MS Gonzalez S Pfeiffer T Olivares S Gonzalez N CD28 costimulation provided through a CD19-specific chimeric antigen receptor enhances in vivo persistence and antitumor efficacy of adoptively transferred T cells. Cancer Res (2006) 66:10995–1004. 10.1158/0008-5472.CAN-06-0160 17108138

[115] Loskog A Giandomenico V Rossig C Pule M Dotti G Brenner MK. Addition of the CD28 signaling domain to chimeric T-cell receptors enhances chimeric T-cell resistance to T regulatory cells. Leukemia (2006) 20:1819–28. 10.1038/sj.leu.2404366 16932339

[116] Milone MC Fish JD Carpenito C Carroll RG Binder GK Teachey D Chimeric receptors containing CD137 signal transduction domains mediate enhanced survival of T cells and increased antileukemic efficacy in vivo. Mol Ther (2009) 17:1453–64. 10.1038/mt.2009.83 19384291 PMC2805264

[117] Savoldo B Ramos CA Liu E Mims MP Keating MJ Carrum G CD28 costimulation improves expansion and persistence of chimeric antigen receptor-modified T cells in lymphoma patients. J Clin Invest (2011) 121:1822–6. 10.1172/JCI46110 21540550 PMC3083795

[118] Wang J Jensen M Lin Y Sui X Chen E Lindgren CG Optimizing adoptive polyclonal T cell immunotherapy of lymphomas, using a chimeric T cell receptor possessing CD28 and CD137 costimulatory domains. Hum Gene Ther (2007) 18:712–25. 10.1089/hum.2007.028 17685852

[119] Maus MV June CH. Making better chimeric antigen receptors for adoptive T-cell therapy. Clin Cancer Res (2016) 22:1875–84. 10.1158/1078-0432.CCR-15-1433 27084741 PMC4843171

