# Supplementary material for: CD137+CD154− Expression As a Regulatory T Cell (Treg)-Specific Activation Signature for Identification and Sorting of Stable Human Tregs from In Vitro Expansion Cultures
Source: Front Immunol. 2018 Feb 7;9:199. doi: 10.3389/fimmu.2018.00199 (PMC5808295; doi:10.3389/fimmu.2018.00199)
Supplement: Supplementary file 1 [file Data_Sheet_1.docx]

Supplementary Material

**CD137+CD154- Expression as a Treg-specific Activation Signature for Identification and Sorting of Stable Human Tregs from In Vitro Expansion Cultures**

**Anna Nowak^1^, Dominik Lock^2^, Petra Bacher^3^, Thordis Hohnstein^3^, Katrin Vogt^4^, Judith Gottfreund^5^, Pascal Giehr^5^, Julia K. Polansky^6^, Birgit Sawitzki^4^, Andrew Kaiser^2^, Jörn Walter^5^, Alexander Scheffold^1,3^***

^1^ German Rheumatism Research Centre (DRFZ) Berlin, Leibniz Association, Berlin, Germany

^2^ Miltenyi Biotec GmbH, Bergisch Gladbach, Germany

^3^ Department of Cellular Immunology, Clinic for Rheumatology and Clinical Immunology, Charité - University Medicine, Berlin, Germany

^4^Institute for Medical Immunology, Charité - University Medicine, Berlin, Germany

^5^ Department of Genetics/Epigenetics, Saarland University, Saarbrücken, Germany

^6^ Berlin-Brandenburg Center for Regenerative Therapies (BCRT), Charité - University Medicine, Berlin, Germany

*** Correspondence:**Alexander Scheffold

[Alexander.Scheffold@charite.de](mailto:Alexander.Scheffold@charite.de)

# Supplementary Figures and Tables

**Supplementary Table 1:** Primer sequences for the detection of Dextran-CAR constructs.

| Construct | Primer | Sequence |
| --- | --- | --- |
| CD137-CD3ζ CAR | CD137 fwd  CD137 rev | CTTCCCGGAGGAAGAAGAGG  CAAGGTTCAGCTCGTTGTAG |
| CD3ζ CAR | CD3ζ fwd  CD3ζ rev | TGTACTGCCTGCAGCGAGTC  TATCCAGCACGTCGTATTCC |
| ICOS-CD3ζ CAR | ICOS fwd  CD3ζ rev | CTGACCGATGTGACACTGAG  TATCCAGCACGTCGTATTCC |
| CD28-CD3ζ CAR | CD28 fwd  CD28 rev | CTGTACTGCCTGCAGAGAAG  GGTGCGTATGGCTGGTAATG |
| CD134-CD3ζ CAR | OX40 fwd  CD3ζ rev | CTGGCCAAGATCAGTACTCG  TATCCAGCACGTCGTATTCC |
| Housekeeping | GAPDH fwd  GAPDH rev | AGGGCTGCTTTTAACTCTGGT  CTCCTCCCACACCAGCTTTG |

**Supplementary Table 2:** Bisulfite-specific primers.

| Target | Amplicon location (Human GRCh37/hg19) | Forward  (5’ 🡪 3’) | Reverse  (5’ 🡪 3’) |
| --- | --- | --- | --- |
| *ctla4* | chr2:204700499-  204700960(+) | GTAGTTGTATGTATTTATTTATTTAAATTTT | CCTTTAATACAAACCAATCTATC |
| *foxp3* | chrX:49117051-  49117387(+) | TGTTTGGGGGTAGAGGATTT | TATCACCCCACCTAAACCAA |
| *ikzf2* | chr2:213988928-  213989329(+) | TATGGGTGTATGTTTTTGTGTGT | CATTACATAACATATCCACTTATAC |
| *il2ra* | chr10:6079456-  6079933(+) | TATTTGTATTTAGTGAAGATTAGAATAA | CTTCTAATTTAAATTTCCCAAAAAAAC |
| *lrrc32* | chr11:76379042-  76379386(+) | TTTTTAGTGAGTATAGTATTAAGTTTTT | CTATAATCAAAACCAAAATTAAAATAAC |
| *cd40lg* | chrX:135739037-  135739484(+) | ATTTTGTAAAGATTAATAGGTTTTAAGA | CATAATAATAATAACTATCCATATATTAC |
| *tnfrsf9* | chr1:8000840-  8001383(+) | TAGATTTTGAGATTTTAGGGTTG | TCATACCAAATTCTAAAATTCTTTC |

## Supplementary Figures


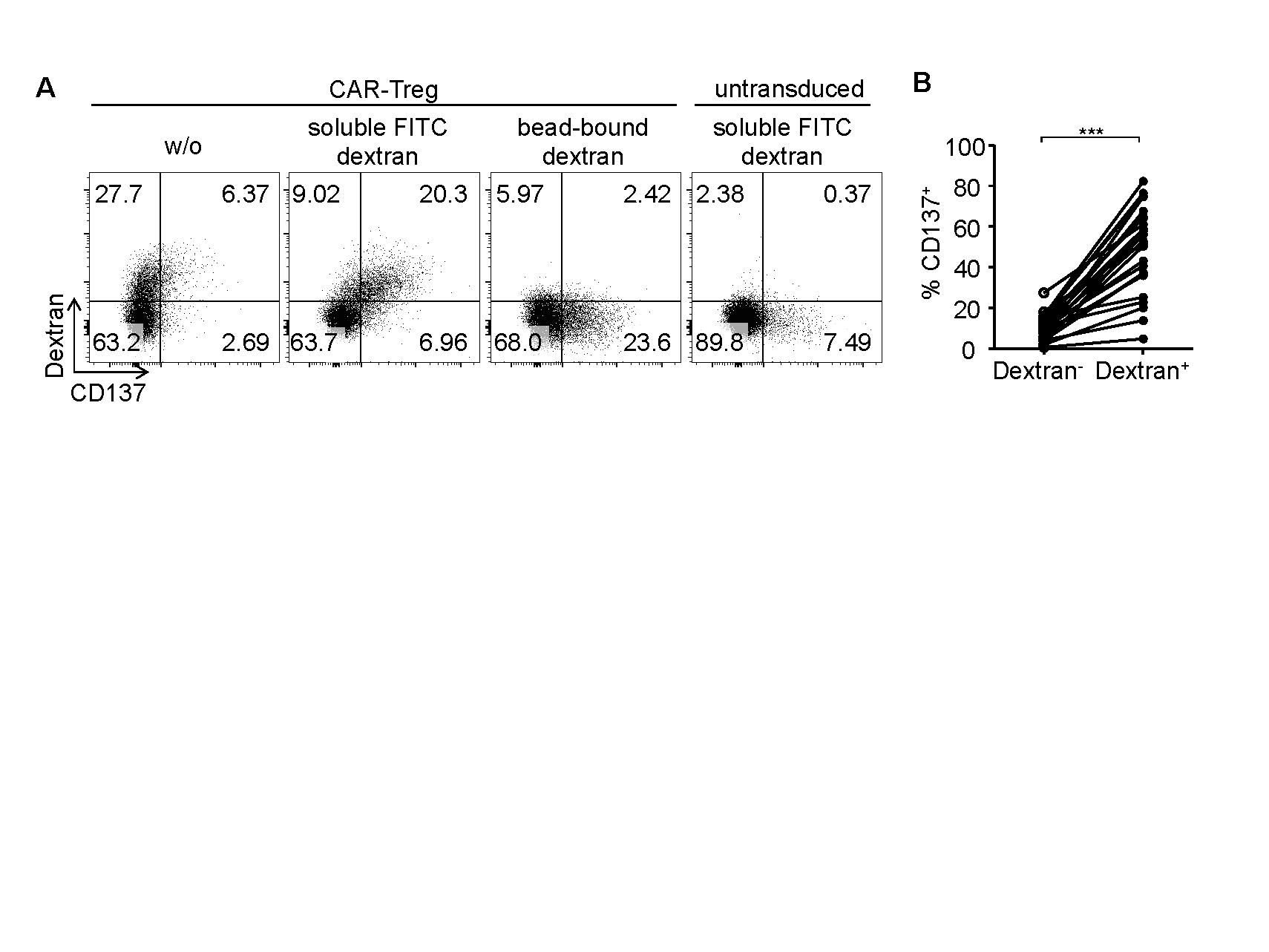


Supplementary Figure 1: Activation of CAR-Tregs. **(A,B)** CD137 expression was analysed on dextran+ and dextran- cells after 6h stimulation with FITC-Dextran. **(A)** Representative dot plot of one donor and **(B)** statistical summary of several donors (n=25, 9 independent experiments were performed). **(B)** Each dot represents one donor, statistical significance was determined by paired t test.


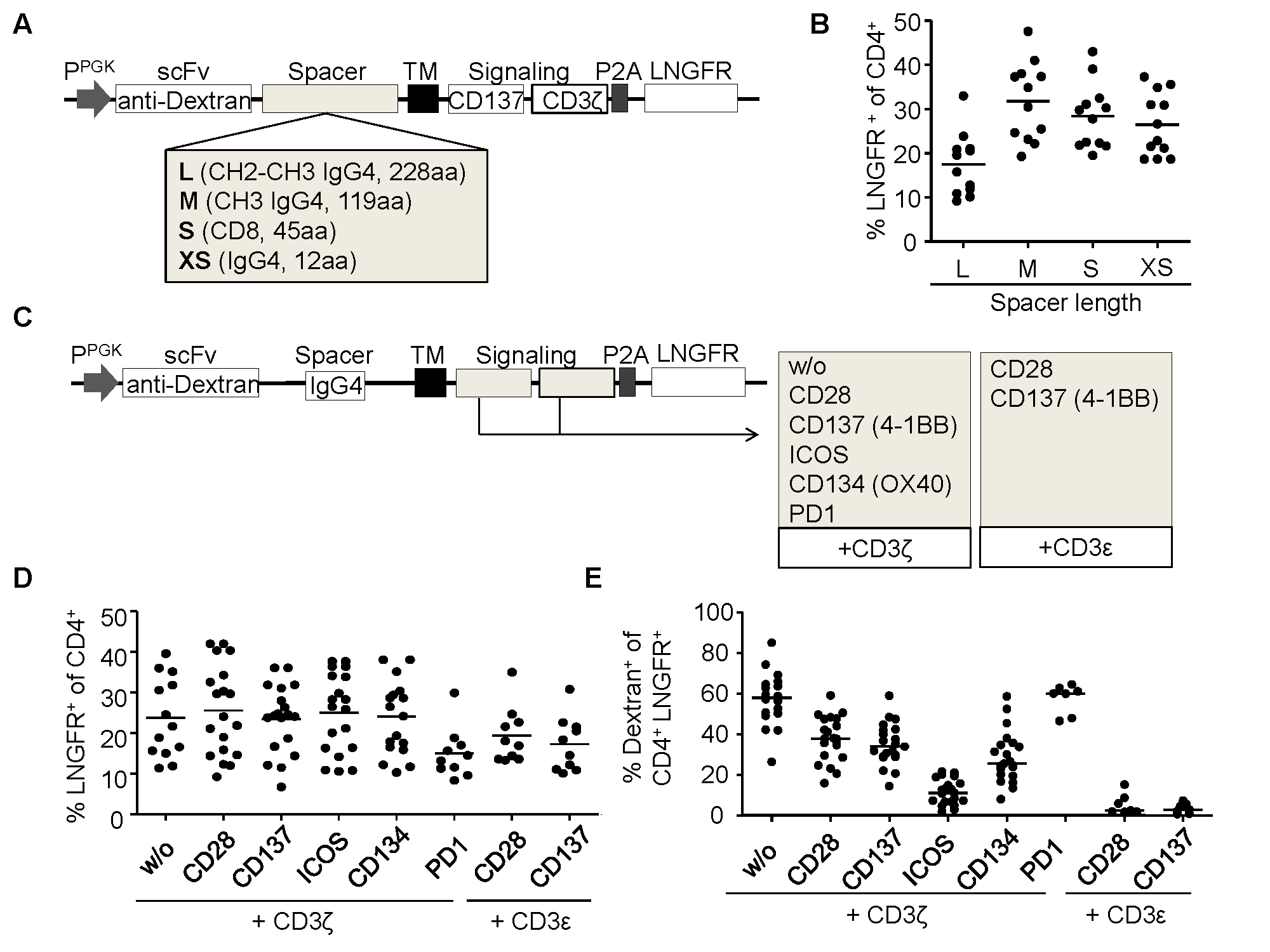


Supplementary Figure 2: Generation of CAR-Tregs with different spacer and signaling domains. **(A-B)** CAR-Tregs with different spacer domains were generated. **(A)** Schematic diagram of the different constructs and **(B)** LNGFR expression after transduction of CD25+ Tregs are shown (n=12 from 4 independent experiments). **(C-E)** CAR-Tregs with different signaling domains were generated. **(C)** Schematic diagram of the different constructs and **(D)** LNGFR expression (n=10-19 from 3-6 different experiments) and **(E)** dextran-binding (n=7-21 from 2-7 independent experiments) after transduction of CD25+ Tregs are shown. **(D,E)** Each dot represents one donor and lines indicate the **(D)** mean or **(E)** median.
